# Quantifying Time-Dependent Predictors for the International Spatial Spread of Highly Pathogenic Avian Influenza H5NX: Focus on Trade and Surveillance Efforts

**DOI:** 10.1155/tbed/2020766

**Published:** 2025-05-08

**Authors:** Lina Awada, Bram Vrancken, Julien Thézé, Christian Ducrot, Paolo Tizzani, Simon Dellicour, Alice Fusaro, Karine Chalvet-Monfray

**Affiliations:** ^1^World Organisation for Animal Health, Paris 75017, France; ^2^Université Clermont Auvergne, INRAE, VetAgro Sup, UMR EPIA, Saint-Genès-Champanelle, France; ^3^KU Leuven, Department of Microbiology, Immunology and Transplantation, Division of Clinical and Epidemiological Virology, Rega Institute for Medical Research, Leuven, Belgium; ^4^Spatial Epidemiology Lab (SpELL), Université Libre de Bruxelles, Brussels, Belgium; ^5^Univ Montpellier, UMR ASTRE, CIRAD, INRAE, Montpellier, France; ^6^Istituto Zooprofilattico Sperimentale delle Venezie (IZSVe), Viale dell'Università 10, Legnaro 35020, Italy

**Keywords:** highly pathogenic avian influenza, migratory birds, phylogeography, poultry trade, surveillance and preventive measures, transboundary spread

## Abstract

The multiple waves of intercontinental transmission of the highly pathogenic avian influenza (HPAI) H5Nx Gs/GD lineage since its identification in 1996 are testament to its resistance to control and prevention efforts. Knowledge of the predictors of HPAI international spread can help identify strengths as well as areas for improvement in surveillance and controlling HPAI. We used 10 years of data with quarterly granularity from the World Organization for Animal Health (WOAH), United Nations (UN), International Union for Conservation of Nature (IUCN), and genetic databases for 2.3.2.1c and 2.3.4.4b H5Nx clades, to determine the impact on international viral spread of (1) six categories of poultry commodities of legal international trade, (2) wild birds' migration, (3) five types of preventive measures, (4) resources allocated to veterinary services, and (5) geographic distance between countries. Two analytical approaches were used: a generalized linear mixed model (GLMM) for all targeted countries, based on epidemiological, trade, and bird migration data. Then, phylogeography-informed generalized linear models (GLMs) with time-dependent predictors were specified for analyzing the HPAI spread between countries with available genetic data. The main conclusions of this study are that results suggested (1) a role of poultry trade in disease spread; (2) a role of migratory birds in disease spread; (3) a strong role of proximity between countries in disease spread; (4) a protective effect for resources allocated to veterinary services; and (5) a protective effect for precautions at borders in exposed countries (protective against informal trade). Our findings show the importance of proper implementation of preventive measures, as advocated in WOAH standards. In addition, our results show the complementarity of epidemiological, trade, biological, and genetic data to trace back international H5NX spread.

## 1. Introduction

Highly pathogenic avian influenza (HPAI) is cause3d by influenza A viruses of the family Orthomyxoviridae. Since its identification in China in 1996, there have been multiple waves of intercontinental transmission of the H5Nx Gs/GD lineage virus [1–3]. HPAI has resulted in the death and mass slaughter of nearly 556 million poultry worldwide between January 2005 and December 2023, with peaks or more than 141 million losses in 2022, 83 million in 2023, 72 million in 2021, and 40 million in 2016 [3, 4]. In addition, as of April 2024, humans have been infected with subtypes H5N1 (around 880 cases in the world), H7N9 (more than 1500 cases), H5N6 (around 90 cases), H9N2 (around 125 cases reported), and sporadic cases have been reported with subtypes H3N8, H6N1, H7N4, H7N7 and H10N3, H10N7, H10N8 [5–10].

One main pathway of HPAI international spread is through the movement of wild birds, as migratory birds can carry avian influenza viruses asymptomatically over long distances [11–14]. Trade of live poultry infected with HPAI, or of poultry products contaminated with HPAI viruses may occur and constitute the other main pathway of HPAI international spread [15–20].

Moreover, several studies suggest that policies and certain characteristics of trading countries may protect against the risk of HPAI introduction, in particular (a) surveillance and early warning, (b) biosecurity and precaution at borders, (c) distance between countries, (d) competencies of veterinary services, and (e) proper implementation of international trade standards [18, 19, 21–27].

Phylogeography is the study of the principles and processes related to the geographical distribution of lineages that constitute genetically related populations [28]. When the evolution and spatial spread of pathogens occur on the same timescale, the history of spread through time can be reconstructed starting from geo- and temporally annotated molecular sequences [29]. Generalized linear models (GLMs), with time-dependent predictors and coefficients, can be used as an extension of phylogeographic inference to inform pathogen spread rates between discrete geographic locations [30–33 ]. Phylogeography coupled with GLM has been used to analyze predictors of HPAI spread, at local, national, or supranational level in China, Egypt, Europe, France, Indonesia, and USA [34–43]. It has also been used to analyze predictors of LPAI spread H9N2 at the international level [44, 45].

It is recognized that influenza viruses, with the vast reservoir of waterfowl, are impossible to eradicate [7]. Furthermore, the zoonotic nature and pandemic potential of avian influenza viruses make their introduction into new countries particularly worrying [46]. A better understanding of the predictors of HPAI international spread through time will help identify strengths as well as areas for improvement in the approach of veterinary services to surveillance and control. Toward this end, the objectives of the article were (1) identifying at-risk flows between affected countries, focusing on the different trade commodities and migratory bird movements and (2) highlighting the characteristics of exposed and source countries which best explain the viral spread.

In this study, we used 10 years of data analyzed at the international level for H5Nx clades 2.3.2.1c and 2.3.4.4b, to determine the impact on international viral spread, with high temporal granularity (quarter year), of (1) six categories of poultry commodities of legal international trade, (2) wild birds' migration movements of 39 species, (3) five types of preventive and surveillance measures implemented by national veterinary services, (4) resources allocated to veterinary services, and (5) geographic distance between countries. These two clades were selected because they each reached at least Africa, Asia, and Europe.

First, a generalized linear mixed model (GLMM) was run for all targeted countries, based on epidemiological, temporal, trade, and bird migration data only. Then, phylogeo-coupled GLM with time-dependent coefficients was run on a subset of affected countries.

## 2. Materials and Methods

### 2.1. Countries Targeted in the Analysis, Disease Introduction Data, and Viral Genetic Data

According to the Global Initiative on Sharing Avian Influenza Data (GISAID, https://www.gisaid.org/), the global network of expertise on animal influenzas (OFFLU, https://www.offlu.org/) of the World Organization for Animal Health (WOAH), and the Food and Agriculture Organization (FAO) of the United Nations (UN) and the literature, 31 countries were affected by clade 2.3.2.1c (H5N1 subtype) between the third quarter of 2009 and second quarter of 2017. The viruses circulated mainly in western Africa, Asia (south-eastern, eastern, southern and western), and eastern Europe. According to the same sources, 55 countries were affected by clade 2.3.4.4b (subtypes H5N1, H5N2, H5N5, H5N6, and H5N8) between the first quarter of 2013 and the first quarter of 2018. The viruses circulated mainly in Europe (southern, western, and northern Europe [47–57]).

**Figure 1:**
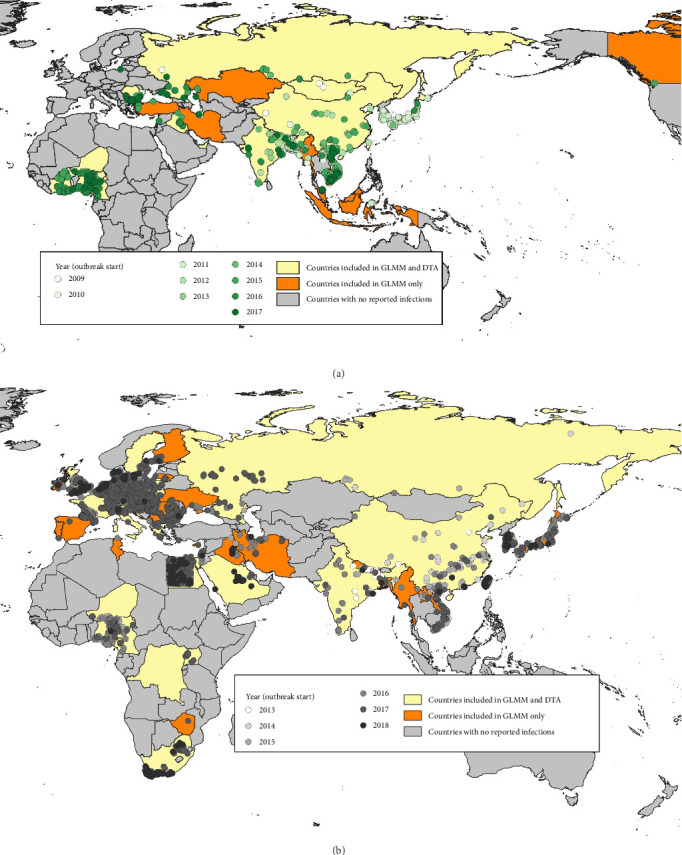
HPAI outbreaks notified to WOAH in countries targeted in the analysis. Each dot corresponds to an outbreak compatible with the clades' timeframes and locations. (a) is for clade 2.3.2.1c and (b) is for clade 2.3.4.4b. Geolocation of outbreaks in Egypt for clade 2.3.4.4b was generated randomly within the country since this information was not provided to WOAH.

Outbreak data (grouped by events) submitted by national authorities to WOAH did not have clade information, in the timeframe considered for this analysis. Dates and locations of (i) the events reported to WOAH and (ii) the positive samples described in the sources mentioned above were compared. Based on that, we selected the outbreaks compatible with the clades studied in this analysis. When several clades were cocirculating, the same outbreaks were considered compatible with each of the cocirculating clades. According to data submitted by national authorities to WOAH, these countries/territories reported, for the period of analysis, about 2300 outbreaks compatible with clade 2.3.2.1c and about 6500 outbreaks compatible with clade 2.3.4.4b (Figure 1). Based on this data, for each clade and each subtype in each country, the first reporting of HPAI was considered as the first introduction. Also, based on HPAI seasonality, reporting after at least 1 year of absence was considered as re-introduction. The list of introductions/reintroductions defined according to these criteria was used as the binary-dependent variable in the GLMM described below. All the countries affected by at least one of the two clades were included in the GLMM analysis.

For phylogeographic inference (discrete trait analysis [DTA]—see details below) coupled with a GLM, the viral genetic data of the HA segment were retrieved from the epi-based dataset used by Fusaro et al. [49]. Starting from a collection of HA genes for which the number of sequences by geographic area under study was generally proportional to the number of reported outbreaks in that geographic area, Fusaro et al. [49] applied three different subsampling procedures to the sequence data in order to mitigate sampling bias. As it was found that the overall migration pattern is consistent across the downsampling strategies, we opt to use the selection of representative sequences by different epidemiological characteristics that have the most balanced distribution of samples among locations and hosts. For further details on the data set compilation strategy, we refer the reader to the Supporting Information provided by Fusaro et al. [49]. It corresponds to 23 countries/territories for clade 2.3.2.1c and 28 countries for clade 2.3.4.4b (Figure 1).

### 2.2. Poultry Trade Flows

An exercise was first organized with selected international experts to achieve consensus on poultry and poultry products likely to spread HPAI through international trade, and they recommended to consider the following categories: (1) more than 1-day-old chickens, turkeys, quails, guinea fowls, pheasants, ducks, geese, ostriches, emus (highest likelihood); and (2) chickens, turkeys, quails, guinea fowls, pheasants, ducks, geese, ostriches, emus of 1-day-old or after hatching (lower likelihood). United Nations (UN) Comtrade [58] centralizes data on international trade, provided by exporting and importing countries and territories. Data were extracted for the period from 2009 to 2018, for the countries and territories in Africa, Asia, and Europe, and for Canada with a final update as of April 10, 2020. For the same trade exchange, when information on price and quantity was provided by both exporting and importing countries, the data provided by the importing country were considered more accurate [59]. Quarterly data for chickens, ducks, geese, guinea fowls, and turkeys were available for the period between 2010 and 2018. In the article, based on the categories available in Comtrade, these animals were stratified into the following subcategories: “chicken lighter than 185 g”, “poultry other than chicken lighter than 185 g”, “chicken of 185 g or more”, and “poultry other than chicken of 185 g or more”. Quarterly data for hatching eggs were only available for the period between 2012 and 2018 and were grouped under the names “chicken hatching eggs” and “hatching eggs of poultry other than chicken”. Annual data for live poultry were available for the entire study period.

Estimation of missing quarterly quantity data for the period from 2009 to 2018 was performed as follows: 1) when only the monetary value was available, the estimate of missing quantity data was performed using the methods described in Awada et al. [60]. (2) Moreover, when only a yearly value was available, the estimate of missing quarterly data was performed based on the quarterly average of quantity percentage traded between the same pair of countries, for the same poultry subcategory, during other years. In case the pair of countries had not traded the same poultry subcategory in other years, the yearly quantity was equally distributed among all quarters. (3) Finally, to estimate missing quarterly data for hatching eggs from 2009 to 2011 (no data available in Comtrade), the average of quantities traded between countries during other quarters of the analysis (between 2012 and 2018) was computed, and the best fitting regression of the values through time was exponential (*R*^2^ = 0.80). Therefore, the exponential function was applied for quantities traded between each pair of countries during other quarters. Such corrections were used in previous publications [60] and are consistent with methods described by the United Nations Conference on Trade and Development [61], as well as with the method later developed in the context of the UN Comtrade upgrade plan in 2019 [62]. In the models described below, trade quantities were centered and scaled. The regression residuals were normally distributed after this transformation.

The transformation of quantitative variables into centered and scaled variables enables the effects of different predictors to be compared, independently of the quantitative units of the different variables.

### 2.3. Migratory Bird Flows

First, the main migratory wild bird species potentially involved in HPAI international spread were identified. As mentioned above, national authorities provide WOAH, through its World Animal Health Information System (WAHIS), information on diseases including the species affected in disease outbreaks [3]. The list of wild bird species affected by HPAI was extracted from WAHIS. Out of these species, only the ones that were (i) categorized as migratory, (ii) whose range overlaps the main study area (Africa, Asia, or Europe) and (iii) reported as affected by HPAI in at least two countries, were considered as potentially involved in HPAI international spread. For 35 species, all these criteria were fulfilled. The list was completed by four additional high-risk waterfowl species listed in a review paper [63]. The final list of the 39 wild bird species potentially involved in HPAI international spread is presented in Tables S1 and **S2**. Second, breeding and nonbreeding sites of the 39 wild bird species listed above in the geographical scope of interest were extracted from the International Union for Conservation of Nature (IUCN) website [64], and each country of interest was tagged for having or not having breeding or nonbreeding sites. Finally, for each species, a table with two columns was constructed. All possible pairs of country breeding site/country with nonbreeding were included in the table. The temporality of the flows was estimated based on the corresponding migratory periods in both directions (based on a literature review and expert advice; Table **S1**). To estimate the size of migratory bird populations in flows, this dataset was finally compiled into a matrix, weighted with the sum of the average centered and scaled global population estimates for species migrating during a given quarter, between 2009 and 2018 (details are available in Table **S2**). The migration periods and global population estimates during a given semester were considered stable across the years, not taking into consideration variations due to changing environmental and climatic conditions.

### 2.4. Preventive and Surveillance Measures Implemented by Veterinary Services Over Time and Resources Allocated to Veterinary Services

Surveillance and preventive measures data were obtained from WOAH. These data are submitted to WAHIS by the national authorities of 183 Member Countries that have the legal obligation to report information concerning high-impact animal diseases listed by WOAH, and more than 20 additional countries and territories that provide information on a voluntary basis [3]. This annual list of diseases contains about 120 diseases, of which HPAI [65]. Six-monthly data were extracted for the period from 2009 to 2018, for the 67 countries and territories targeted by the analysis, with a final update as of April 10, 2020. Measures implemented during a given semester were considered valid for both quarters within this time range. These data were for (a) active surveillance in poultry for HPAI (including targeted surveillance and screening, as defined by WOAH); (b) passive surveillance in poultry for HPAI (including disease official notification, general surveillance, and monitoring, as defined by WOAH); (c) any surveillance in wild birds for HPAI (including all the measures listed above); (d) precautions at borders against HPAI, and (e) preventive vaccination against HPAI (considering that, based on previous studies, vaccinated poultry can also be a source of spread, as vaccination can in some cases mask clinical signs without preventing infection [19, 66, 67]). Based on the WOAH definition, precautions at borders include “measures applied at airports, ports, railway stations or road checkpoints open to international movement of animals, animal products and other related commodities, where import inspections are performed to prevent introduction of the disease, infection or infestation into a country/territory or zone” [3]. The variables used for these predictors were binary (“1” when implemented and “0” when not implemented), in each country and for a given quarter. Ten percent of the data were missing in WOAH information systems and were estimated, based on information provided for the previous years and subsequent years, with the hypothesis that countries and territories maintain some consistency in the surveillance and control policies implemented. Additionally, gross domestic product (GDP) per capita was used as a proxy for the resources allocated to veterinary services. Data were extracted from the World Bank [68]. In the models described below, GDP per capita was centered and scaled. The regression residuals were normally distributed after this transformation.

### 2.5. GLMM for Disease Introduction and Reintroduction

For each of the two clades considered in each country, the list of introductions/reintroductions defined in Section 2.1 was used as the binary dependent variable. The detailed description of the GLMM for disease introduction and reintroduction is presented in Section S1.

The full model was tested for each clade, then variables were removed progressively, comparing the values of the Akaike information criterion (AIC), preferring the model with the lowest AIC value. The final model was selected for each clade according to the AIC criterion. After this step, collinearity between statistically significant predictors was tested with variable inflation factors (VIFs), and no collinearity issue was detected. A GLMM approach was selected because our data consisted of repeated measures of imports from countries over time. R version 3.5.1. and package “glmmTMB” were used for the GLMM, with *i* (country exposed) as a random variable and “binomial” distribution family [69, 70]. The dependent variable, predictor variables, and units used in the model are summarized in Figure S1a.

### 2.6. Phylogeographic Inference Coupled With GLM With Time-Dependent Coefficients for Viral Spread

We performed a Bayesian phylogeographic inference using the discrete diffusion model [71], which is also referred to as a DTA. For each clade, a set of 1000 evenly spaced post-burn-in phylogenies were selected from the epianalyses reported by Fusaro et al. [49]. These served as empirical tree distributions [32] for reconstructing the history of spread in discrete space under a time variable GLM [31] in BEAST v.1.10 [72] with BEAGLE v.3 [73] for improved computational speed. The GLM model was coupled to a model averaging procedure (Bayesian stochastic search variable selection, BSSVS) that provides a test to identify significant predictors. To ensure that the Bayes factor (BF) support estimates are not driven by the relative abundances of samples by location, the BF support was estimated with the default (BF_default_, [32]) as well as the adjusted (BF_adjusted_, [74]) setup. Maximum clade credibility (MCC) summary trees were obtained with TreeAnnotator 1.10 distributed with BEAST v1.10 and visualized with FigTree [75]. Convergence and mixing properties were inspected using Tracer v.1.7 [76]. The detailed description of the GLM coupled with phylogeographic inference with time-dependent coefficients for viral spread is presented in Section S2.

The periods covered by the predictors are 2009–2017 for clade 2.3.2.1 and 2013–2018 for clade 2.3.4.4. The dependent variable, predictor variables, and units used in the model are summarized in Figure S1b.

Effect size (with lower and upper 95% highest posterior density interval estimates) is a measure of the size of mean differences or strength of associations. BF adjusted of 25 was considered as cutoff for significance. After this step, collinearity between predictors with BF_adjusted_ equal to or higher than 25 was tested with VIF, and no collinearity issue was detected. A positive effect size indicated a positive association (when this variable increases, the spread of HPAI increases), and a negative effect size indicated a negative association (when this variable increases, the spread of HPAI decreases).

### 2.7. Counts of Quarters, Countries, and Number of Sequences for Each Model and Clade

Counts of quarters, countries, and number of sequences for each model and clade are presented in Table S5. For the GLMM, the number of observations was nearly equivalent for clades 2.3.2.1c and 2.3.4.4b. For the phylogeography-informed GLM, the total number of sequences considered for the two clades were comparable. Finally, from the counts of countries sequences by world region, we see that for clade 2.3.2.1c, Asia is the region most represented in the clade distribution (with more than 50% of countries and sequences from this region), and Europe for 2.3.4.4b.

## 3. Results

To illustrate the variability and range distribution of the continuous predictor variables across all countries in the dataset, summary statistics are presented in Table 1.

### 3.1. GLMM for Disease Introduction and Reintroduction

Results of final GLMMs for the two clades are presented in Table S3. First, concerning potential flows of HPAI spread as follows:

1. Significant positive association was found between HPAI introductions/reintroductions and quantities imported for chicken lighter than 185 g for clade 2.3.2.1c (*p* =0.05, OR = 1.13, 95% CI: 1.01–1.28) and clade 2.3.4.4b (*p* = 0.01, OR = 1.20, 95% CI: 1.03–1.40). For both clades, the import of chicken lighter than 185 g was an aggravating factor associated with HPAI introductions/reintroductions.
2. Significant positive association was found between HPAI introductions/reintroductions and quantities imported for hatching eggs of poultry other than chicken for clade 2.3.2.1c (*p* = 0.03, OR = 1.18, 95% CI: 1.01–1.39). For that clade, the import of hatching eggs of poultry other than chicken was an aggravating factor associated with HPAI introductions/reintroductions.
3. Significant positive association was found between HPAI introductions/reintroductions and incoming flow of migratory birds for clade 2.3.4.4b (*p* = 0.001, OR = 1.35, 95% CI: 1.11–1.64). For that clade, the incoming flow of migratory birds was an aggravating factor associated with HPAI introductions/reintroductions.
4. Lastly, significant strong positive association was found between HPAI introductions/reintroductions and proximity with affected countries for the two clades, capturing informal trade, wild birds' movements across short distances, and human movements (for clade 2.3.2.1c: *p* = 0.004, OR = 1.54, 95% CI: 1.14–2.10 and for clade 2.3.4.4b: *p* < 0.001, OR = 2.01, 95% CI: 1.44–2.79). For both clades, proximity was an aggravating factor associated with HPAI introductions/reintroductions.

Second, concerning preventive and surveillance measures implemented in countries:

1. This study found no significant association between HPAI introductions/reintroductions and (a) the percentage of connected affected source countries reporting passive poultry surveillance, (b) the percentage of connected affected source countries reporting active poultry surveillance, (c) the percentage of connected affected source countries reporting surveillance in wild birds, (d) the percentage of connected affected source countries reporting preventive poultry vaccination, or (e) GDP per capita in exposed and connected affected source countries, used as proxy for the resources allocated to veterinary services.
2. Negative association was found between HPAI introductions/reintroductions and precautions at borders in exposed countries for clade 2.3.2.1c (*p* < 0.001, OR = 0.31, 95% CI: 0.17–0.58) and clade 2.3.4.4b (*p* = 0.01, OR = 0.54, 95% CI: 0.33–0.88). For both clades, precautions at borders were a protective factor associated with HPAI introductions/reintroductions.

Finally, concerning temporal variables:

1. This study found no significant association between HPAI introductions/reintroductions and years. This shows that there was no significant effect of year-specific predictors other than those included in the model.
2. For both clades, negative association was found between HPAI introductions/reintroductions and quarter 3 (July–September) (for clade 2.3.2.1c, *p* < 0.001, OR = 0.21, 95% CI: 0.06–0.71 and for clade 2.3.4.4b, *p* =0.05, OR = 0.42, 95% CI: 0.17, 1.00). This means that fewer introductions/reintroductions occurred between July and September. For clade 2.3.4.4b, positive association was found with quarter 4 (October–December; *p* = 0.03, OR = 1.72, 95% CI: 1.04–2.85). This means that most introductions/reintroductions occurred between October and December for this clade.

### 3.2. Phylogeographic Inference Coupled With GLM With Time-Dependent Coefficients for Viral Spread

As explained above, HPAI viral migration was only analyzed for countries associated with sufficient viral sequences after the subsampling step by Fusaro et al. [49]. The evolutionary relationship between viral lineages from these countries is reported through the spatially annotated time-scaled phylogenetic tree obtained through the DTA and displayed in Figure S2.

For the GLM, results for the factors that influence HPAI spread between countries for each of the clades of interest are presented in Table S4.

First, concerning potential flows of HPAI spread are as follows:

1. High BF and positive values for effect size were measured for one poultry trade flow (chicken hatching eggs) for clade 2.3.4.4b (BF_adjusted_ = 73.51, effect size = 0.15 [0.08, 0.27]). BFs were rather low for clade 2.3.2.1c. For clade 2.3.4.4b, import of chicken-hatching eggs was an aggravating factor associated with HPAI spread.
2. BFs were rather low for migratory birds for the two clades.
3. Very high BF and strong negative values for effect size were measured for distance for the two clades (which captures informal trade, wild birds' movements across short distances and human movements): for clade 2.3.2.1c: BF_adjusted_ = 153,874, effect size = −2.24 [−3.15, −1.4]; for clade 2.3.4.4b: BF_adjusted_ = 494,450, effect size = −1.14 [−1.60, −0.67]. For both clades, distance was a protective factor associated with HPAI spread.

Second, concerning preventive and surveillance measures implemented in countries:

1. BFs were low for precautions at borders in exposed countries (maximum BF_adjusted_ = 4.42).
2. High BF and negative values for effect size were measured for clade 2.3.4.4 b for a) GDP per capita in source countries (BF_adjusted_ = 63.22, effect size = −0.85 [−1.43, −0.26]) and b) GDP per capita in exposed countries, (BF_adjusted_ = 48.31, effect size = −0.40 [−0.69, −0.18]). Both were used as proxies for the resources allocated to veterinary services. For clade 2.3.4.4b, increased resources were a protective factor associated with HPAI spread.
3. High BF and positive values for effect size were measured for clade 2.3.4.4b for active surveillance in poultry in source countries (BF_adjusted_ = 91.98, effect size = 1.63 [0,47, 2.81]). For clade 2.3.4.4b, active surveillance in poultry in source countries was surprisingly an aggravating factor associated with HPAI spread.

Comparative results of (a) the GLMM for disease introduction and reintroduction and (b) phylogeography-informed GLM are presented in Table S6.

## 4. Discussion

This is the first study on HPAI combining two methods (GLMM and phylogeo-coupled GLM with time-dependent coefficients) over such a broad geographical and temporal range and with such temporally detailed predictors. The main conclusions suggested by this study are as follows: (1) a role of poultry trade in disease introduction/reintroduction and disease spread; (2) a role of migratory birds in disease introduction/reintroduction for clade 2.3.4.4b; (3) a major effect of proximity between countries (capturing informal trade, short-distance movements of wild birds and human movements) in the introduction/reintroduction and spread of diseasel (4) aa protective effect for disease introduction/reintroduction and disease spread for GDP in source and exposed countries (proxy for resources allocated to veterinary services)—however, these associations have only been identified for one clade and by one method. (5) Similarly, a protective effect for disease introduction/reintroduction and disease spread for precautions at borders in exposed countries (protective against inform trade). Finally, (6) for two clades, time effects of quarters were suggested by results obtained.

In our study, the role of poultry trade (hatching eggs and chicken lighter than 185 g) in disease introduction/reintroduction and disease spread was suggested by the results obtained. The stratification of the trade variable on poultry species (chicken vs., other species) and age (hatching eggs, poultry lighter than 185 g and poultry of 185 g or more) was done with the intention to identify with more accuracy trade categories likely to have had the biggest impact. Using phylogenetic relationships between virus isolates at the peak of the first H5N1 panzootic wave (clade 2.2 between 2003 and 2005), Kilpatrick et al. [17] estimated that about 20% of the viral spread events studied worldwide were due to international trade, and that spread through trade was mainly observed in Asia, but very few events in Europe and Africa were associated with this pathway. In our study, Asia is the most represented region in the distribution of clade 2.3.2.1c, and Europe is the most represented region in that of clade 2.3.4.4b. The significance of the commercial factors was demonstrated for both clades, with comparable odds ratios for the GLMM. Also, in the case of H5N1 clade 2.2, previous studies have shown significant phylogenetic clustering in Southeast Asia, which was consistent with the high frequency of live animal movements between countries in this region because of increasing intraregional trade [20, 77], and the long-distance spread of HPAI H5N1 viruses in 2004 was found to be caused by movements of domestic poultry [16]. These previous studies suggest that HPAI spread through poultry trade was common in certain regions of the World such as Asia during the period of global spread of clade 2.2., which could not be included in this study. Also, another study, covering several HPAI clades, has subsequently suggested that the risk of HPAI introduction increased by the importation of live poultry in the same year, which is a methodology very similar to what was proposed in ours for the GLMM [18]. However, this study did not include countries' proximity, and in this context, trade can be a confounding factor since countries preferably trade poultry with countries of the same world Region [60]. In the analysis presented in this article, it was important to include proximity between countries to eliminate confounding bias and confirm associations with trade. Although several examples of cross-border spread of HPAI through trade have been described in the literature, these events seem to be rare [19, 78–81].

Our GLMM analysis confirmed that migratory birds' flows increased viral spread for the two clades of interest. Previous studies have established that avian influenza virus lineages can spread along migratory routes [82–87] and phylogeographic analyses have previously shown the effects of transport of HPAI H5N1 by different bird species across Asia [88]. In our analysis, for all clades, Russia and China were the largest countries, and by far the countries with the most population size at origin and destination, due to a variety of species having breeding and nonbreeding sites within these two countries. The sites within these countries may play an important role for HPAI spread through migratory birds' pathway.

• Furthermore, our analysis showed that proximity between countries is associated with viral spread for the two clades. This predictor potentially captures informal trade, wild birds' movements across short distances, and human movements between countries. Few studies have been conducted on the role of informal or illegal movements in HPAI spread. However, several examples in different regions of the world seem to prove its importance. These examples include the 2016 HPAI event in Lebanon in connection with the massive influx of refugees and several HPAI events in Asia and Africa in in 2003/2004 and 2006. Fighting cocks had probably introduced the disease into Malaysia [16, 17, 19, 79, 89]. Most of these examples suggest that the importance of informal trade for disease spread would be relevant between neighboring countries. Another interesting result of our study is the significant negative association measured for both clades between HPAI introduction/reintroduction and precautions at borders, defined by WOAH as ‘measures applied at airports, ports, railway stations, or road check-points open to international movement of animals, animal products, and other related commodities, where import inspections are performed to prevent the introduction of the disease, infection, or infestation into a country/territory or zone'. This is also in favor of a role of informal trade in HPAI spread, although this was not found with the phylogeographic inference coupled with GLM. Informal trade is not taken into account in official import data (and therefore the trade variables used in this study). However, precautions at borders can theoretically reduce the probability of virus introduction via this informal route. The negative association highlighted in this study is consistent with this hypothesis: the more precautions at borders, the lower the probability of introduction. Also, some previous studies have shown the role of short-distance migratory Anseriforms (e.g., mallard ducks) in the spread of clade 2.3.4.4 in Asia [90], which is also captured in our proximity variable.
• Our study did not identify any negative association between surveillance and disease introduction/reintroduction, or disease spread. Some previous studies however suggested that the risk of cross-border spread of HPAI through trade depends on the surveillance capacity of the importing country [19, 27]. Indeed, where infectious disease surveillance and laboratory resources are limited, many cases are likely to go undetected [18]. Thus, to be effective, veterinary surveillance must be rigorous and target all possible routes of spread [19]. It is possible that poor-quality data used for surveillance predictors may have had an impact on the results obtained. The surveillance data used was purely binary (presence or absence of surveillance) but did not reflect the quality of the surveillance implemented. Surprisingly, for poultry, active surveillance in source countries for clade 2.3.4.4b was positively associated with disease spread. This is likely to be a confusion bias, since countries affected by HPAI are more likely to implement surveillance compared with nonaffected countries, and countries implementing active surveillance are more likely to detect disease introduction than others.
• Besides, previous studies suggested that the use of vaccination against avian influenza in poultry could reduce the severity of symptoms and thus delay detection in a flock [91]. However, our study did not find a significant association between a preventive vaccination in poultry in source countries and HPAI introductions/reintroductions or HPAI spread. This may be due to the low number of countries reporting vaccination during the study period in the three target countries/territories. These countries/territories included Bangladesh, China, Egypt, Germany, Hong Kong, Indonesia, Kazakhstan, Laos, Mongolia, Niger, Russia, and Vietnam [3].
• In our study, GDP per capita in source and exposed countries (proxy for resources allocated to veterinary services) was negatively associated with disease spread for clade 2.3.4.4b. This is consistent with several previous studies, which demonstrated associations between economic indices and HPAI introduction. This is explained by the correlation between the level of economic development and the capacity of veterinary services in surveillance, alert, general biosecurity, and HPAI control [18, 23, 26, 27].

For both clades, negative association was found between HPAI introductions/reintroductions and quarter 3, and for clade 2.3.4.4b, positive association was found with quarter 4. These associations capture temporal effects resulting from avian hosts' ecology, virus properties, and climatic variables. Previous studies described HPAI global seasonality, consistent with this finding [4]. Variables related to avian hosts' ecology, virus properties, and climatic variables, such as temperature, humidity, and precipitation [36, 81, 92–97] were not included in the model, which mainly focused on trade and surveillance/prevention efforts.

• Concerning predictors of HPAI spread at the global level, most studies have been using as dependent variable disease presence data in countries, and rarely viral migration data between countries, which is more accurate. Previous studies based on GLM coupled with phylogeographic data have all been performed on smaller geographical regions. Considering that the epidemiology of HPAI is complex and affected by many local factors, the main objective of this study was to identify predictors that play a role at global scale, in the same way across Africa, Asia, and Europe and across years. It does not capture factors that are specific to smaller areas. For example, the effect of trade predictors can vary between regions and periods because of disparities in implementation of sanitary and phytosanitary measures (SPS), World Trade Organization (WTO), and WOAH standards [27], but this is not reflected in our analysis. Considering this, added values of the analysis consist in fine granularity of data for predictors (categories of poultry trade and surveillance/preventive measures); high temporal granularity (quarter year); and the use of two different methods (GLMM and phylogeography-informed GLM), which make the results robust.
• A main limitation of this study relates to data quality. As highlighted in Fusaro et al. [49], the variability in wild bird surveillance efforts among countries and in time generates underdetection of HPAI cases in wild birds and limit our ability to infer their contribution in the spatial movements of the virus. Also, trade and epidemiological data used are based on official reporting, which has limitations due to the variability among countries in national customs institution, veterinary services, and other national systems. Additionally, the poultry commodities are grouped into categories that are based on UN Harmonized Commodity Description and Coding Systems and are not risk-based [98]. This categorization does not allow to isolate day-old chicks, which weight about 40 g, and have a lower risk of disease transmission than other live poultry according to the experts and literature [99, 100]. Finally, a proxy was built to estimate wild birds' migration since no accurate and complete data are available, and flows were considered stable per quarter across all years of the analysis, which constitutes a simplification that leads to a loss of power in the models.
• Another limitation consists in the low numbers of affected countries that could be integrated into the analyses by phylogeographic inference coupled with GLM, due to limited sequence availability. Some sequences may have only recently been published and have not been included in the dataset analyzed, and for some countries, the clade information may be available from technical reports, although no genetic data has been published. We must also take account of the fact that there may be countries for which no information on clades is available for a certain period. Yet, we were able to run the phylogeography-informed GLM on high estimated percentages of affected countries for clade 2.3.21c (74% of affected countries, for which genetic data was available), although the percentage was much lower for clade 2.3.4.4b (51%).
• Results should be interpreted considering the geographical distributions of the two clades. For clade 2.3.2.1c, Asia is the region most represented in the clade distribution, and Europe for 2.3.4.4b. These geographical disparities may explain some differences observed between the results for those two clades. It would be interesting to explore further, through other studies, how differences between regions, such as the abundance and distribution of host species, commercial, cultural and political contexts and poultry consumption habits may affect the associations between the various predictors studied and the spread of HPAI.
• Each model used in this analysis has added value and limitations. First, the GLMM allows to include countries considered affected but applies to disease introduction/reintroduction and not to viral spread. Also, disease introduction/reintroduction events are based on estimates and may not accurately represent the real situation. In the model, data for potential source countries of the virus were aggregated, for each quarter and each exposed country. It would have been possible to include the data in the model separately for each source country, but this would have required the addition of the source country as another random variable, making the model more cumbersome, with potential convergence difficulties. Second, the phylogeography-informed GLM applies to disease spread, which is more relevant to the research question but could only include a limited estimated proportion of affected countries (51% to 74% for each clade) due to limited sequence availability, as presented above. In addition, this method presents a bias in estimates of viral spread rates when sampling is heterogenous across subpopulations since the phylogeographic model assumes that sample sizes across subpopulations are proportional to subpopulation sizes [101]. The impact of this bias should be nuanced though, since it can be alleviated by increasing the sample size and balancing spatial and temporal composition in the samples [102]. In our study, sampling heterogeneity between countries is likely to result in bias in the phylogenetic trees. Despite the methodological limitations described, phylogeography-informed GLM results of the inferred epidemics dynamics are consistent with previous studies [1, 103–105], which gives some confidence to the results of the GLM.

Our findings show the importance of proper implementation of surveillance measures, as advocated in the standards of WOAH [65]. WOAH standards, recognized by the WTO under the SPS Agreement for animal health and zoonoses-related matters, contribute effectively to global regulatory approaches to safeguard public good while limiting unnecessary impediments. In addition, our analysis shows the complementarity of epidemiological, trade, biological, and viral genetic data to trace back international viral spread and understand its predictors. Interconnection of such databases could be very useful for controlling the international spread of animal diseases.

This analysis provides an interesting insight into the recent global situation to identify strengths as well as areas for improvement in the approach of national authorities to controlling HPAI globally. To complete this analysis, it would be interesting to better map international informal trade, since our study suggests its role in viral spread, based on the distance proxy. Moreover, increased sampling and sequencing efforts can help to improve our understanding of the means and routes of virus diffusion. Finally, similar approaches can be applied to other clades, other areas, or smaller areas to show specificities of viral spread in other contexts.

## Figures and Tables

**Table 1:** Summary statistics of continuous predictor variables used to inform the models describing HPAI spread.

Clade	Variable name	Mean	Standard deviation	Range
2.3.2.1c	Monthly import of chicken hatching eggs from another country included in the analysis (kg)	380	10,166	0–891,577
	Monthly import of chicken lighter than 185 g from another country included in the analysis (kg)	542	10,515	0–437,062
	Monthly import of chicken of 185 g or more from another country included in the analysis (kg)	18	950	0–79,550
	Monthly import of hatching eggs of other poultry from another country included in the analysis (kg)	16	383	0–27,137
	Monthly import of other poultry lighter than 185 g from another country included in the analysis (kg)	99	3404	0–158,557
	Monthly import of other poultry of 185 g or more from another country included in the analysis (kg)	49	1491	0–102,985
	Migratory birds (index for population size)	138	234	18–2,551
	Geographic distance from another country included in the analysis (km)	6246	3662	216–13,520
	GDP per capita of countries included in the analysis	9650	13,681	390–48,603

2.3.4.4b	Monthly import of chicken hatching eggs from another country included in the analysis (kg)	83,151	681,953	0–14,210,593
	Monthly import of chicken lighter than 185 g from another country included in the analysis (kg)	196,894	2,010,803	0–60,613,721
	Monthly import of chicken of 185 g or more from another country included in the analysis (kg)	254,760	3,500,204	0–95,305,869
	Monthly import of hatching eggs of other poultry from another country included in the analysis (kg)	2834	21,591	0–657,666
	Monthly import of other poultry lighter than 185 g from another country included in the analysis (kg)	18,724	159,253	0–3,134,711
	Monthly import of other poultry of 185 g or more from another country included in the analysis (kg)	9278	98,109	0–2,531,168
	Migratory birds (index for population size)	158	212	18–2551
	Geographic distance from another country included in the analysis (km)	4724	3020	189–13,172
	GDP per capita of countries included in the analysis	20,017	16,764	457–53,044

## Data Availability

The data that support the findings of this study are available from the corresponding author upon reasonable request.

## Supporting Information

Supporting Information 1 Figure S1: Dependent variable, predictor variables, and units used in each analysis. Figure S1a is for the generalized linear mixed model (GLMM). Figure S1b is for inferred dispersal history of viral lineages based on a discrete traits analysis coupled with generalized linear models (DTA-GLM)

Supporting Information 2 Figure S2: Inferred dispersal history of viral lineages based on a discrete traits analysis coupled with generalized linear models (DTA-GLM) visualized by a time-scaled maximum clade credibility (MCC) summary phylogeny representing the evolutionary relationships between the sampled viral lineages. Figure S1a is for clade 2.3.2.1c. Figure S1b is for clade 2.3.4.4b.

Supporting Information 3 Table S1: Migratory periods of wild bird species potentially involved in HPAI international spread

Supporting Information 4 Table S2: Global population estimation of wild bird species potentially involved in HPAI international spread

Supporting Information 5 Table S3: Results of final GLMMs regressing HPAI first introduction/reintroduction in the exposed country on quantity of poultry commodities traded, incoming migratory birds, characteristics of exposed and source countries and temporality.

Supporting Information 6 Table S4: Results of GLM coupled with DTA regressing HPAI spread between countries on quantity of poultry commodities traded, incoming migratory birds and characteristics of exposed and source countries.

Supporting Information 7 Table S5: Counts of quarters, countries, and sequences for each model and clade.

Supporting Information 8 Table S6: Comparative results of a) the GLMM for disease introduction and reintroduction, and b) phylogeography-informed GLM.

Supporting Information 9 Section S1: Detailed description of the GLMM for disease introduction and reintroduction.

Supporting Information 10 Section S2: Detailed description of the GLM coupled with phylogeographic inference with time-dependent coefficients for viral spread.
