# Supplementary material for: Quantifying Time-Dependent Predictors for the International Spatial Spread of Highly Pathogenic Avian Influenza H5NX: Focus on Trade and Surveillance Efforts
Source: Transbound Emerg Dis. 2025 May 8;2025:2020766. doi: 10.1155/tbed/2020766 (PMC12643678; doi:10.1155/tbed/2020766)
Supplement: Supporting Information 1 — Figure S1: Dependent variable, predictor variables, and units used in each analysis. Figure S1a is for the generalized linear mixed model (GLMM). Figure S1b is for inferred dispersal history of viral lineages based on a discrete traits analysis coupled with generalized linear models (DTA-GLM) [file 2020766.f1.docx]

**
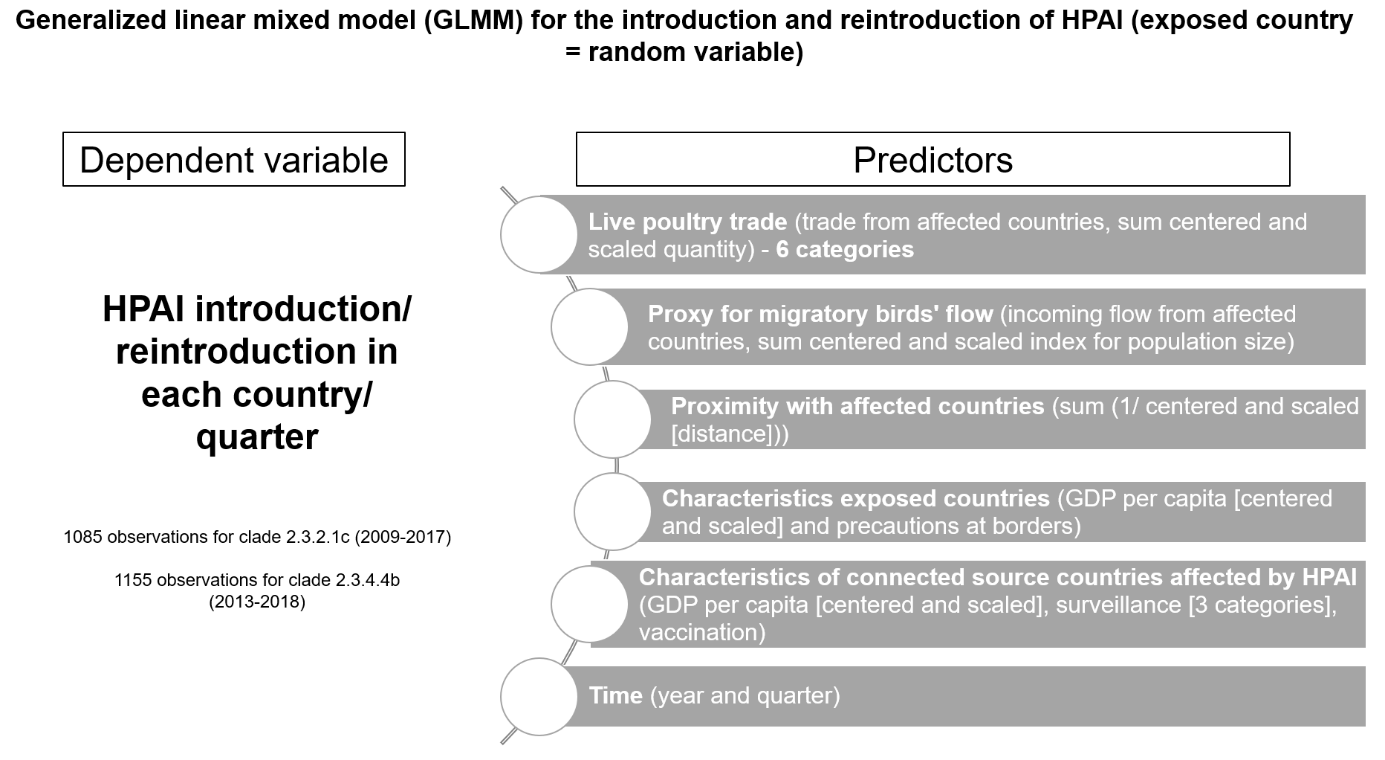
**

**Figure S1a.**

**
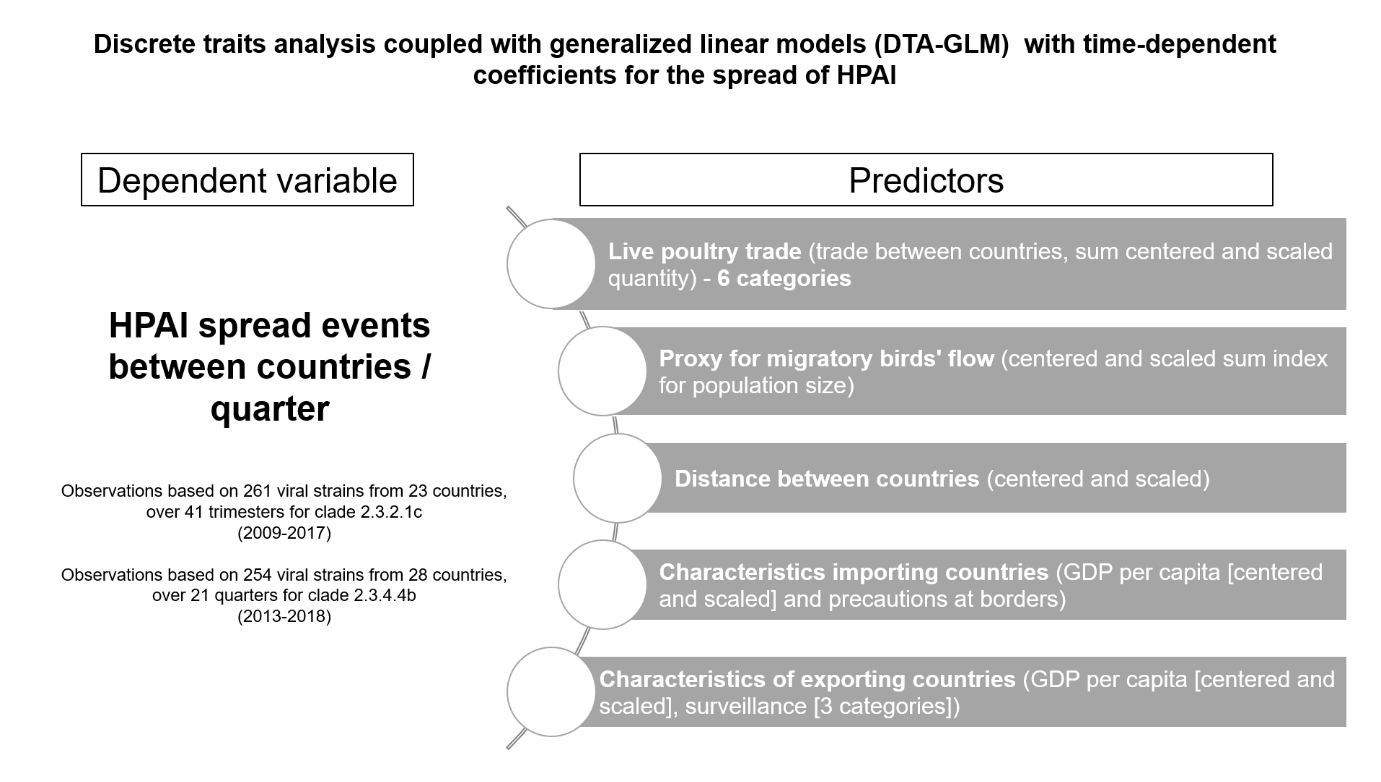
**

**Figure S1b.**

**Figure S1.** Dependent variable, predictor variables and units used in each analysis. Figure S1a is for the generalized linear mixed model (GLMM). Figure S1b is for inferred dispersal history of viral lineages based on a discrete traits analysis coupled with generalized linear models (DTA-GLM)
