# Supplementary material for: Quantifying Time-Dependent Predictors for the International Spatial Spread of Highly Pathogenic Avian Influenza H5NX: Focus on Trade and Surveillance Efforts
Source: Transbound Emerg Dis. 2025 May 8;2025:2020766. doi: 10.1155/tbed/2020766 (PMC12643678; doi:10.1155/tbed/2020766)
Supplement: Supporting Information 2 — Figure S2: Inferred dispersal history of viral lineages based on a discrete traits analysis coupled with generalized linear models (DTA-GLM) visualized by a time-scaled maximum clade credibility (MCC) summary phylogeny representing the evolutionary relationships between the sampled viral lineages. Figure S1a is for clade 2.3.2.1c. Figure S1b is for clade 2.3.4.4b. [file 2020766.f2.docx]

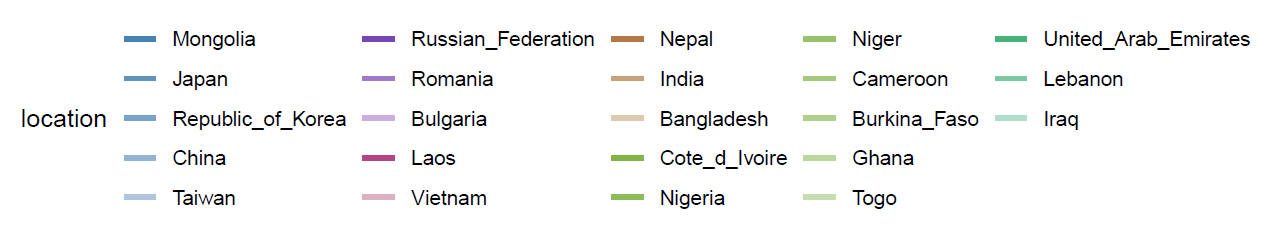


*
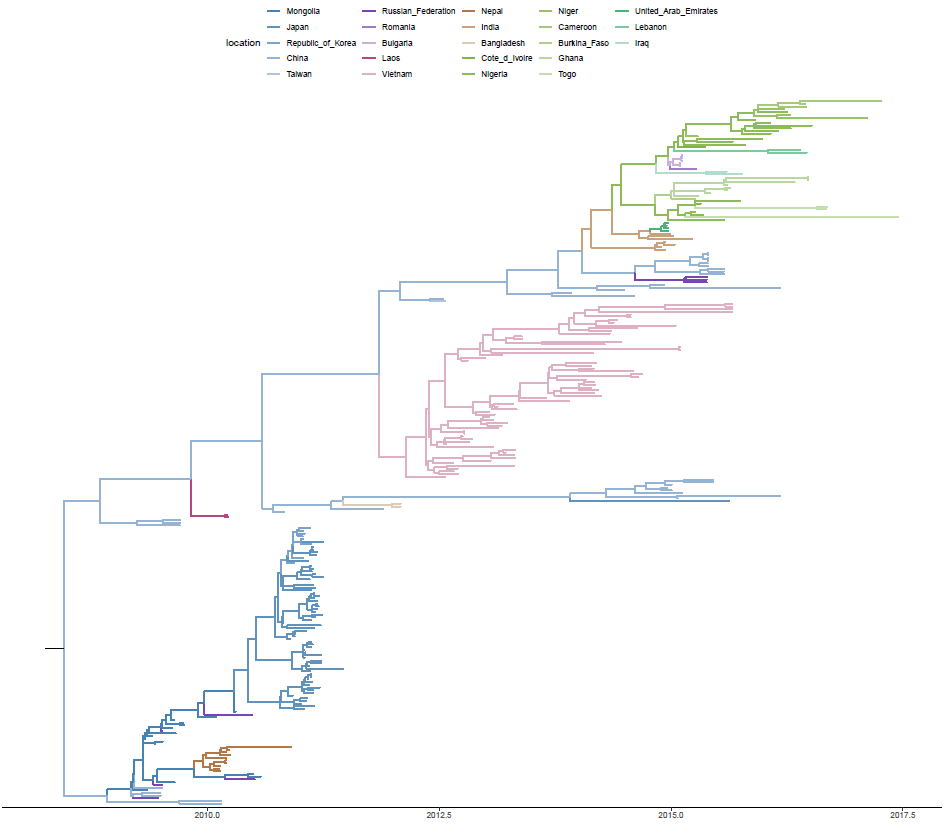
*

**Figure S2a.**

**
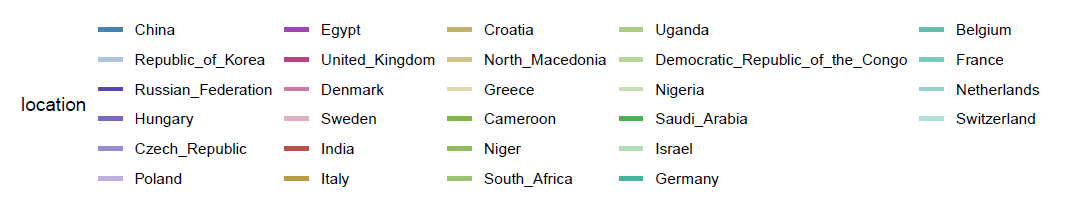
**

**
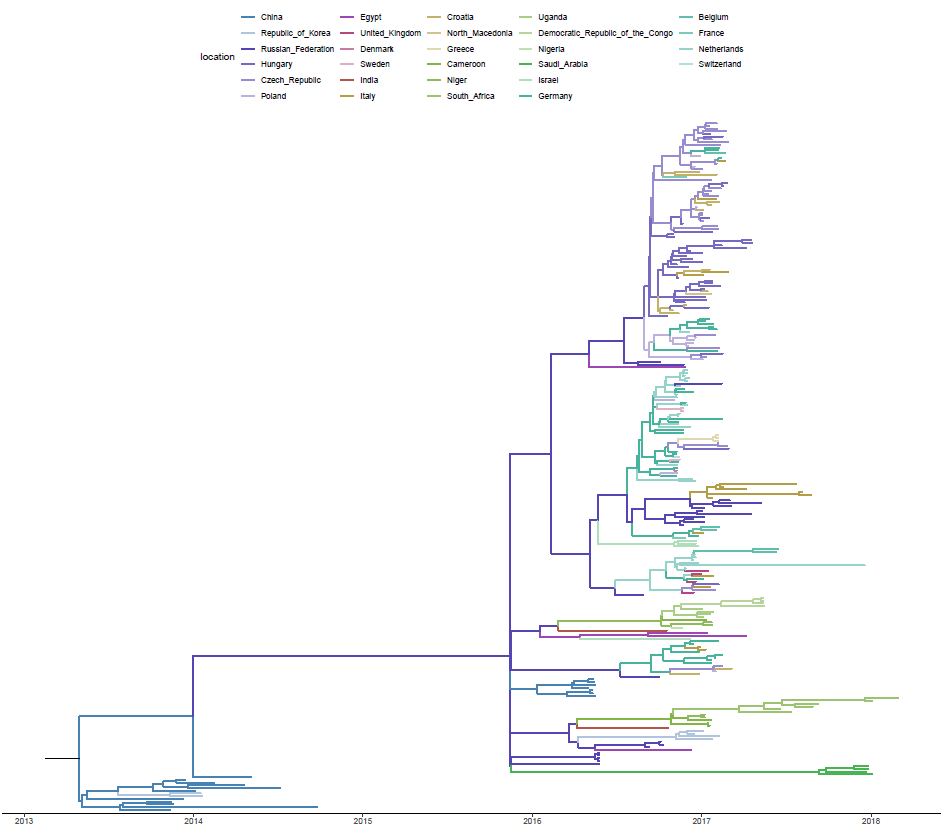
**

**Figure S2b.**

**Figure S2.** Inferred dispersal history of viral lineages based on a discrete traits analysis coupled with generalized linear models (DTA-GLM) visualized by a time-scaled maximum clade credibility (MCC) summary phylogeny representing the evolutionary relationships between the sampled viral lineages. The color of a branch indicates the inferred location (see legend) with the highest posterior support. A change of colors indicates a virus’ migration spread event. Figure S1a is for clade 2.3.2.1c. Figure S1b is for clade 2.3.4.4b. Gradients of the same colors are used for countries of the same world regions. based on UN Geographical regions described in https://unstats.un.org/unsd/methodology/m49/)
