# Supplementary material for: Quantifying Time-Dependent Predictors for the International Spatial Spread of Highly Pathogenic Avian Influenza H5NX: Focus on Trade and Surveillance Efforts
Source: Transbound Emerg Dis. 2025 May 8;2025:2020766. doi: 10.1155/tbed/2020766 (PMC12643678; doi:10.1155/tbed/2020766)
Supplement: Supporting Information 3 — Table S1: Migratory periods of wild bird species potentially involved in HPAI international spread [file 2020766.f3.docx]

**Table S1.** Migratory periods of wild bird species potentially involved in HPAI international spread

| **Species** | **Migratory period from non-breeding sites to breeding sites** | **Migratory period from breeding sites to non-breeding sites** | **References** |
| --- | --- | --- | --- |
| *Accipiter Gentilis* | February to April | September to November | Ferguson-Lees, James; Christie, David A. (2001). Raptors of the World. Houghton Mifflin Harcourt. ISBN 978-0-618-12762-7.; Squires, J. R. Ruggiero (1995). "Winter movements of adult northern goshawks that nested in southcentral Wyoming". J. Raptor Res. 29: 5–9.; Atkinson, E. C.; Goodrich, L. J.; Bildstein, K. L. (1996). "Temporal field guide to autumn raptor migration at Hawk Mountain Sanctuary, Pennsylvania". Pennsylvania Birds. 10: 134–137.; Smith, G. A.; Muir, D. G. (1980). "Derby Hill spring hawk migration". Birding. 12: 224–234. |
| *Anas acuta* | March to May | August to October | Delany, S., Veen, J. & Clark, J.A. (eds) 2006. Urgent preliminary assessment of ornithological data relevant to the spread of Avian Influenza in Europe. Report to the European Commission. Study contract: 07010401/2005/425926/MAR/B4. Authors: Atkinson, P.W., Clark, J.A., Delany, S., Diagana, C.H., du Feu, C., Fiedler, W., Fransson, T., Gaulthier-Clerc, M., Grantham, M.J., Gschweng, M., Hagemeijer, W., Helmink, T., Johnson, A., Khomenko, S., Martakis, G., Overdijk, O., Robinson, R.A., Solokha, A., Spina, F., Sylla, S.I., Veen, J. & Visser, D. http://ec.europa.eu/environment/nature/nature_conservation/focus_wild_birds/avian_influenza/index_en.htm |
| *Anas bahamensis* | January to June | July to December | Expert advice |
| *Anas crecca* | February to March | August to December | Giunchi, D.; Baldaccini, N.E.; Lenzoni, A.; Luschi, P.; Sorrenti, M.; Cerritelli, G.; Vanni, L. (2019). "Spring migratory routes and stopover duration of satellite‐tracked Eurasian Teals Anas crecca wintering in Italy". Ibis. 161 (2): 117–130. doi:10.1111/ibi.12602;  Birdweb: <http://birdweb.org/birdweb/bird/green-winged_teal>; Animal diversity web : http://animaldiversity.org/accounts/Anas_crecca/classification/ |
| *Anas penelope* | March to August | September to November | Clements, James, (2007) The Clements Checklist of the Birds of the World, Cornell University Press, Ithaca; Dunn, J. & Alderfer, J. (2006) National Geographic Field Guide to the Birds of North America 5ª Ed.; Floyd, T. (2008) Smithsonian Field Guide to the Birds of North America Harper Collins, NY; |
| *Anas plathyrhynchos* | January to June | July to December | Expert advice |
| *Anas strepera* | February to March | September to February | SUEUR, F. & TRIPLET, P. (1999).- Les oiseaux de la Baie de Somme. Inventaire commenté des oiseaux de la Baie de Somme et de la Plaine Maritime Picarde. SMACOPI, Groupe Ornithologique Picard, Conservatoire Littoral, Réserve Naturelle de la Baie de Somme. 510 p.; KHARITONOV, S.P. (2002).- Migration and some population parameters of the Gadwall Anas strepera in Europe and North Asia. In NOSKOV, G.A., CZAJKOWSKI, A. & FERTIKOVA, K. P. (Eds).- Study of the Status and Trends of Migratory Bird Populations in Russia (4th Iss.). OMPO Sp. Publ. World & Family, St. Petersburg. 123-142 p.; |
| *Anser albifrons* | March to May | August to October | Delany, S., Veen, J. & Clark, J.A. (eds) 2006. Urgent preliminary assessment of ornithological data relevant to the spread of Avian Influenza in Europe. Report to the European Commission. Study contract: 07010401/2005/425926/MAR/B4. Authors: Atkinson, P.W., Clark, J.A., Delany, S., Diagana, C.H., du Feu, C., Fiedler, W., Fransson, T., Gaulthier-Clerc, M., Grantham, M.J., Gschweng, M., Hagemeijer, W., Helmink, T., Johnson, A., Khomenko, S., Martakis, G., Overdijk, O., Robinson, R.A., Solokha, A., Spina, F., Sylla, S.I., Veen, J. & Visser, D. http://ec.europa.eu/environment/nature/nature_conservation/focus_wild_birds/avian_influenza/index_e  n.htm |
| *Anser anser* | February to March | September to December | Greylag Goose ( Anser anser ) movements" (PDF). British Trust for Ornithology. Retrieved 24 October 2015. stated to be from Delany, S.; Veen, J.; Clark, J.A., eds. (2006). Urgent preliminary assessment of ornithological data relevant to the spread of Avian Influenza in Europe. Report to the European Commission. Study contract: 07010401/2005/425926/MAR/B4. |
| *Anser fabalis* | February to March | September to October | Base communale de la biodiversité et la géodiversité Hauts-de-France : <http://www.donnees.picardie.developpement-durable.gouv.fr/patnat/espic/index.php?get_idcdnom=2724>; |
| *Anser indicus* | January to June | July to December | Expert advice |
| *Ardea alba* | January to June | July to December | Expert advice |
| *Ardea cinerea* | February | September to October | BirdLife International 2012, Ardea cinerea, su IUCN Red List of Threatened Species, Versione 2020.2, IUCN, 2020. |
| *Aythya ferina* | March to May | July to November | Les Oiseaux d'Europe d'Afrique du Nord et du Moyen-Orient Lars Jonsson - Nathan 1994 - ISBN 2-09-241050-4 Europe et environs ; Les Oiseaux d'Europe Catherine BOUCHAIN, Jean C.ROCHÉ, Frédérik ROCKER - Sitelle France 1996 Europe, CD; Folliot, B., Guillemain, M., Champagnon, J. and Caizergues, A., 2018. Patterns of spatial distribution and migration phenology of common pochards Aythya ferina in the Western Palearctic: a ring-recoveries analysis. Wildlife Biology, 2018(1). |
| *Aythya fuligula* | February to April | September | PAVLOV, D.S. & VIKSNE, J. (1989).- Migrations of Birds of Eastern Europe and Northern Asia. Anseriformes. Moscow, Nauka. 135-173 p. (En russe); NOSKOV, G.A., CZAJKOWSKI, A. & FERTIKOVA, K.P. (2001).- Study of the Status and Trends of Migratory Bird Populations in Russia (Third Issue). OMPO Sp. Publ. Omp. World & Family, St. Petersburg. 159 p.; NOSKOV, G.A., CZAJKOWSKI, A. & FERTIKOVA, K.P. (2002).- Study of the Status and Trends of Migratory Bird Populations in Russia (Fourth Issue). OMPO Sp. Publ. Omp. World & Family, St. Petersburg. 150 p.; |
| *Aythya marila* | March to May | August to September | Les Oiseaux d'Europe d'Afrique du Nord et du Moyen-Orient Lars Jonsson - Nathan 1994 - ISBN 2-09-241050-4 Europe et environs ; Les Oiseaux d'Europe Catherine BOUCHAIN, Jean C.ROCHÉ, Frédérik ROCKER - Sitelle France 1996 Europe, CD; |
| *Buteo buteo* | March to April | September to October | Nature gate: <https://luontoportti.com/fr/t/480/buse-variable> |
| *Ciconia ciconia* | March | August to October | Oiseaux : <https://www.oiseaux.net/oiseaux/cigogne.blanche.html> |
| *Corvus frugilegus* | February to March | October to November | Oiseaux Europe : <http://www.oiseaux-europe.com/Oiseau-7/Corbeau-freux.html> |
| *Cygnus atratus* | January to June | July to September | Expert advice |
| *Cygnus columbianus* | April to May | September to November | Chen, W., Doko, T., Fujita, G., Hijikata, N., Tokita, K.I., Uchida, K., Konishi, K., Hiraoka, E. and Higuchi, H., 2016. Migration of tundra swans (Cygnus columbianus) wintering in Japan using satellite tracking: identification of the Eastern Palearctic flyway. Zoological science, 33(1), pp.63-72. |
| *Cygnus cygnus* | March to May | September to November | BirdLife International (2021) Species factsheet: Cygnus cygnus. Downloaded from http://www.birdlife.org on 29/08/2021. Recommended citation for factsheets for more than one species: BirdLife International (2021) IUCN Red List for birds. Downloaded from http://www.birdlife.org on 29/08/2021. |
| *Cygnus olor* | March to April | September to December | Les Oiseaux d'Europe d'Afrique du Nord et du Moyen-Orient Lars Jonsson - Nathan 1994 - ISBN 2-09-241050-4 Europe et environs ; Les Oiseaux d'Europe Catherine BOUCHAIN, Jean C.ROCHÉ, Frédérik ROCKER - Sitelle France 1996 Europe, CD; |
| *Falco peregrinus* | April to May | September to November | White, C. M., N. J. Clum, T. J. Cade, and W. G. Hunt (2020). Peregrine Falcon (Falco peregrinus), version 1.0. In Birds of the World (S. M. Billerman, Editor). Cornell Lab of Ornithology, Ithaca, NY, USA. https://doi.org/10.2173/bow.perfal.01 |
| *Falco tinnunculus* | April to May | August to September | Paul Géroudet, Les rapaces d'Europe diurnes et nocturnes, Paris, Delachaux et Niestlé, 2013 (ISBN 978-2-603-01958-0) ; Roger Peterson, Guy Mountfort, P.A.D. Hollom, Guide des oiseaux d'Europe, Delachaux et Niestlé S. A., 32, rue de Grenelle, Paris VII, 1957 ; Andrew Village, The Kestrel, Londres, T. & A. D. Poyser, 1990 (ISBN 0-85661-054-2); |
| *Fulica atra* | February to May | August to November | BirdLife International (2021) Species factsheet: Fulica atra. Downloaded from http://www.birdlife.org on 29/08/2021; |
| *Gallinula chloropus* | January to April | September to October | Les oiseaux : <http://www.breizh-oiseaux.fr/pouledeau.php> |
| *Haliaeetus albicilla* | February to April | October to December | ZUCCA, M. & le CMR (2004).- Les observations d’oiseaux migrateurs rares en France. 1er rapport du Comité des Migrateurs Rares (juillet 2000-juin 2001). Ornithos 11(5): 193-213. ; ZUCCA, M. & le CMR (2005).- Les observations d’oiseaux migrateurs rares en France. 2ème rapport du Comité des Migrateurs Rares (janv. 2001-déc. 2002). Ornithos 12(5): 233-260 ; GEROUDET, P. (2000).- Les Rapaces diurnes et nocturnes d'Europe. 7e Edition. Delachaux et Niestlé S.A., Lausanne. 446 p. |
| *Larus argentatus* | January to June | July to September | Expert advice |
| *Larus canus* | January to June | July to September | Expert advice |
| *Larus marinus* | March to April | July to November | Bentz G., 2007, LES OISEAUX DU BORD DE MER , Découverte nature, LPO, Ed. Ouest-France, 32p.;Cadiou B., 2002, LES OISEAUX MARINS NICHEURS DE BRETAGNE , Ed. Biotope, 135p.; Claustre G., Lemoine C., Bourgaut Y., Scott C.W., Gayral P., Cosson J., Loir M., Lusardi C., Tavernier Y., 1997, NATURE EN BORD DE MER , ed. Ouest-France, 196p.; Couzi L., Roques H., 2007, GUIDE DE LA FAUNE ET DE LA FLORE DU LITTORAL MANCHE-ATLANTIQUE , ed. Sud-Ouest, Broché, collection Nature, 167p. |
| *Larus ridibundus* | February to April | July to December | François Sueur, La Mouette rieuse, Éveil éditeur, coll. « Approche », Saint-Yrieix-sur-Charente, 2000, 72 p., (ISBN 978-2840000013). |
| *Melanitta nigra* | February to April | September to November | C. Aulert, « Les stationnements de macreuses (Melanitta) sur le littoral augeron. Biogéographie et environnement », thèse universitaire, Caen, 1997, p. 575; Stanley Cramp et K.E.L. Simmons, The Birds of the Western Palearctic, Oxford, London, New York, Oxford University Press, 1977, 722 p.;  DECEUNINCK, B., MAILLET, N., DRONNEAU, C., WARD, A. & MAHEO, R. (2006).- Dénombrements d'anatidés et de foulques hivernant en France -Janvier 2005. WI / LPO / MEDD. 40 p. ; DECEUNINCK, B., MAILLET, N., WARD, A., DRONNEAU, C. & MAHEO, R. (2005).- Dénombrements d'anatidés et de foulques hivernant en France à la mi-janvier 2004. Wetlands International / LPO / DNP, Rochefort. 41 p. |
| *Mergellus albellus* | February to March | October to November | DECEUNINCK, B. (1997).- Synthèse des dénombrements de canards et de foulques hivernant en France 1967-1995. Ministère de l'Environnement-DNP / LPO / Wetlands International. 97 pp. ; DECEUNINCK, B., MAILLET, N., KERAUTRET, L., DRONNEAU, C. & MAHEO, R. (2003).- Dénombrements d'anatidés et de foulques hivernant en France à la mi-janvier 2002. MEDD / Wetlands International / LPO, Rochefort. 41 pp. ; DECEUNINCK, B., MAILLET, N., KERAUTRET, L., RIOLS, C. & MAHEO, R. (2001).- Dénombrements d'anatidés et de foulques hivernant en France à la mi-janvier 2000. LPO / Wetlands International / DNP. 42 p. |
| *Pelecanus crispus* | March | July to September | IOC World Bird List (v11.1), Gill, F and D Donsker (Eds). 2019.; Dalmatian Pelican (Pelecanus crispus) – BirdLife species factsheet. Birdlife.org (1998-10-03). Retrieved on 2012-08-22. |
| *Phalacrocorax carbo* | March to May | August | BirdLife International (2021) Species factsheet: Phalacrocorax carbo. Downloaded from http://www.birdlife.org on 29/08/2021. |
| *Philomachus pugnax* | February to May | July to September | Vaillancourt, Eric; Prud'Homme, Sophie; Haman, François; Guglielmo, Christopher G.; Weber, Jean-Michel (2005). "Energetics of a long-distance migrant shorebird (Philomachus pugnax) during cold exposure and running". Journal of Experimental Biology. 208 (Pt 2): 317–325. doi:10.1242/jeb.01397 |
| *Podiceps cristatus* | March | August | Nature gate: <https://luontoportti.com/fr/t/737/grebe-huppe> |
| *Porzana pusilla* | March to May | August to September | Base communale de la biodiversité et la géodiversité Hauts-de-France: <http://www.donnees.picardie.developpement-durable.gouv.fr/patnat/espic/index.php?get_idcdnom=3045> |
