# Supplementary material for: Quantifying Time-Dependent Predictors for the International Spatial Spread of Highly Pathogenic Avian Influenza H5NX: Focus on Trade and Surveillance Efforts
Source: Transbound Emerg Dis. 2025 May 8;2025:2020766. doi: 10.1155/tbed/2020766 (PMC12643678; doi:10.1155/tbed/2020766)
Supplement: Supporting Information 4 — Table S2: Global population estimation of wild bird species potentially involved in HPAI international spread [file 2020766.f4.docx]

**Table S2.** Global population estimation of wild bird species potentially involved in HPAI international spread

| **Species** | **Global population estimate** | **Population estimate of mature individuals in the world** | **References** |
| --- | --- | --- | --- |
| *Accipiter Gentilis* | 1.5 x population of mature individuals (based on expert advice) | 1 000 000 – 2 500 000 | BirdLife International. 2016. Accipiter gentilis. The IUCN Red List of Threatened Species 2016: e.T22695683A93522852. https://dx.doi.org/10.2305/IUCN.UK.2016-3.RLTS.T22695683A93522852.en. Downloaded on 01 September 2021. |
| *Anas acuta* | 7 100 000 – 7 200 000 |  | BirdLife International. 2019. Anas acuta. The IUCN Red List of Threatened Species 2019: e.T22680301A153882797. https://dx.doi.org/10.2305/IUCN.UK.2019-3.RLTS.T22680301A153882797.en. Downloaded on 01 September 2021. |
| *Anas bahamensis* | 180 000 – 1 000 000 |  | Janet Kear (2005) Ducks, Geese and Swans: Species accounts (Cairina to Mergus). Oxford University Press, 2005 - Anseriformes - 908 pages |
| *Anas crecca* | 6 600 000 – 7 700 000 |  | BirdLife International. 2019. Anas crecca (amended version of 2016 assessment). The IUCN Red List of Threatened Species 2019: e.T22729717A155455470. https://dx.doi.org/10.2305/IUCN.UK.2016-3.RLTS.T22729717A155455470.en. Downloaded on 01 September 2021. |
| *Anas penelope* | 2 800 000 – 3 300 000 |  | BirdLife International. 2017. Mareca penelope (amended version of 2016 assessment). The IUCN Red List of Threatened Species 2017: e.T22680157A111892532. https://dx.doi.org/10.2305/IUCN.UK.2017-1.RLTS.T22680157A111892532.en. Downloaded on 01 September 2021. |
| *Anas plathyrhynchos* | > 19 000 000 |  | BirdLife International. 2019. Anas platyrhynchos (amended version of 2017 assessment). The IUCN Red List of Threatened Species 2019: e.T22680186A155457360. https://dx.doi.org/10.2305/IUCN.UK.2019-3.RLTS.T22680186A155457360.en. Downloaded on 01 September 2021. |
| *Anas strepera* | 4 300 000 – 4 900 000 |  | BirdLife International. 2016. Mareca strepera. The IUCN Red List of Threatened Species 2016: e.T22680149A86020572. https://dx.doi.org/10.2305/IUCN.UK.2016-3.RLTS.T22680149A86020572.en. Downloaded on 01 September 2021. |
| *Anser albifrons* | 3 100 000 – 3 200 000 |  | BirdLife International. 2016. Anser albifrons. The IUCN Red List of Threatened Species 2016: e.T22679881A85980652. https://dx.doi.org/10.2305/IUCN.UK.2016-3.RLTS.T22679881A85980652.en. Downloaded on 01 September 2021. |
| *Anser anser* | 1 000 000 – 1 100 000 |  | BirdLife International. 2018. Anser anser. The IUCN Red List of Threatened Species 2018: e.T22679889A131907747. https://dx.doi.org/10.2305/IUCN.UK.2018-2.RLTS.T22679889A131907747.en. Downloaded on 01 September 2021. |
| *Anser fabalis* | 680 000 – 800 000 |  | BirdLife International. 2018. Anser fabalis. The IUCN Red List of Threatened Species 2018: e.T22679875A132302864. https://dx.doi.org/10.2305/IUCN.UK.2018-2.RLTS.T22679875A132302864.en. Downloaded on 01 September 2021. |
| *Anser indicus* | 97 000 – 118 000 |  | Liu, D., Zhang, G., Li, F., Ma, T., Lu, J. and Qian, F., A revised species population estimate for the bar-headed goose (Anser indicus) Avian Res, 8: 7, 2017. |
| *Ardea alba* | 1.5 x population of mature individuals (based on expert advice) | 590 000 – 2 200 000 | BirdLife International. 2019. Ardea alba (amended version of 2016 assessment). The IUCN Red List of Threatened Species 2019: e.T22697043A155465940. https://dx.doi.org/10.2305/IUCN.UK.2019-3.RLTS.T22697043A155465940.en. Downloaded on 01 September 2021. |
| *Ardea cinerea* | 790 000 – 3 700 000 |  | BirdLife International. 2019. Ardea cinerea. The IUCN Red List of Threatened Species 2019: e.T22696993A154525233. https://dx.doi.org/10.2305/IUCN.UK.2019-3.RLTS.T22696993A154525233.en. Downloaded on 01 September 2021. |
| *Aythya ferina* | 1 950 000 – 2 250 000 |  | BirdLife International. 2019. Aythya ferina (amended version of 2017 assessment). The IUCN Red List of Threatened Species 2019: e.T22680358A155473754. https://dx.doi.org/10.2305/IUCN.UK.2019-3.RLTS.T22680358A155473754.en. Downloaded on 01 September 2021. |
| *Aythya fuligula* | 2 600 000 – 2 900 000 |  | BirdLife International. 2016. Aythya fuligula. The IUCN Red List of Threatened Species 2016: e.T22680391A86013549. https://dx.doi.org/10.2305/IUCN.UK.2016-3.RLTS.T22680391A86013549.en. Downloaded on 01 September 2021. |
| *Aythya marila* | 4 920 000 – 5 130 000 |  | BirdLife International. 2018. Aythya marila. The IUCN Red List of Threatened Species 2018: e.T22680398A132525108. https://dx.doi.org/10.2305/IUCN.UK.2018-2.RLTS.T22680398A132525108.en. Downloaded on 01 September 2021. |
| *Buteo buteo* | 2 170 000 – 3 690 000 |  | BirdLife International. 2017. Buteo buteo (amended version of 2016 assessment). The IUCN Red List of Threatened Species 2017: e.T61695117A119279994. https://dx.doi.org/10.2305/IUCN.UK.2017-3.RLTS.T61695117A119279994.en. Downloaded on 01 September 2021. |
| *Ciconia ciconia* | 700 000 – 704 000 |  | BirdLife International. 2016. Ciconia ciconia. The IUCN Red List of Threatened Species 2016: e.T22697691A86248677. https://dx.doi.org/10.2305/IUCN.UK.2016-3.RLTS.T22697691A86248677.en. Downloaded on 01 September 2021. |
| *Corvus frugilegus* | 54 300 000 – 94 700 000 |  | BirdLife International. 2017. Corvus frugilegus (amended version of 2016 assessment). The IUCN Red List of Threatened Species 2017: e.T22705983A118782308. https://dx.doi.org/10.2305/IUCN.UK.2017-3.RLTS.T22705983A118782308.en. Downloaded on 01 September 2021. |
| *Cygnus atratus* | 100 000 – 1 000 000 |  | BirdLife International. 2018. Cygnus atratus. The IUCN Red List of Threatened Species 2018: e.T22679843A131907524. https://dx.doi.org/10.2305/IUCN.UK.2018-2.RLTS.T22679843A131907524.en. Downloaded on 01 September 2021. |
| *Cygnus columbianus* | 317 000 – 336 000 |  | BirdLife International. 2016. Cygnus columbianus. The IUCN Red List of Threatened Species 2016: e.T22679862A89644875. https://dx.doi.org/10.2305/IUCN.UK.2016-3.RLTS.T22679862A89644875.en. Downloaded on 01 September 2021. |
| *Cygnus cygnus* | 180 000 |  | BirdLife International. 2016. Cygnus cygnus. The IUCN Red List of Threatened Species 2016: e.T22679856A85965262. https://dx.doi.org/10.2305/IUCN.UK.2016-3.RLTS.T22679856A85965262.en. Downloaded on 01 September 2021. |
| *Cygnus olor* | 598 000 – 615 000 |  | BirdLife International. 2016. Cygnus olor. The IUCN Red List of Threatened Species 2016: e.T22679839A85946855. https://dx.doi.org/10.2305/IUCN.UK.2016-3.RLTS.T22679839A85946855.en. Downloaded on 01 September 2021 |
| *Falco peregrinus* | 140 000 |  | BirdLife International. 2019. Falco peregrinus (amended version of 2016 assessment). The IUCN Red List of Threatened Species 2019: e.T45354964A155500538. https://dx.doi.org/10.2305/IUCN.UK.2016-3.RLTS.T45354964A155500538.en. Downloaded on 01 September 2021. |
| *Falco tinnunculus* | 1.5 x population of mature individuals (based on expert advice) | 4 000 000 – 6 500 000 | BirdLife International. 2016. Falco tinnunculus. The IUCN Red List of Threatened Species 2016: e.T22696362A93556429. https://dx.doi.org/10.2305/IUCN.UK.2016-3.RLTS.T22696362A93556429.en. Downloaded on 01 September 2021 |
| *Fulica atra* | 7 950 000 – 9 750 000 |  | BirdLife International. 2019. Fulica atra. The IUCN Red List of Threatened Species 2019: e.T22692913A154269531. https://dx.doi.org/10.2305/IUCN.UK.2019-3.RLTS.T22692913A154269531.en. Downloaded on 01 September 2021. |
| *Gallinula chloropus* | 2 900 000 – 6 200 000 |  | BirdLife International. 2019. Gallinula chloropus (amended version of 2016 assessment). The IUCN Red List of Threatened Species 2019: e.T62120190A155506651. https://dx.doi.org/10.2305/IUCN.UK.2019-3.RLTS.T62120190A155506651.en. Downloaded on 01 September 2021. |
| *Haliaeetus albicilla* | 1.5 x population of mature individuals (based on expert advice) | 20 000 – 49 999 | BirdLife International. 2020. Haliaeetus albicilla. The IUCN Red List of Threatened Species 2020: e.T22695137A181768148. https://dx.doi.org/10.2305/IUCN.UK.2020-3.RLTS.T22695137A181768148.en. Downloaded on 01 September 2021. |
| *Larus argentatus* | 2 060 000 – 2 430 000 |  | BirdLife International. 2018. Larus argentatus. The IUCN Red List of Threatened Species 2018: e.T62030608A132672776. https://dx.doi.org/10.2305/IUCN.UK.2018-2.RLTS.T62030608A132672776.en. Downloaded on 01 September 2021. |
| *Larus canus* | 2 500 000 – 3 700 000 |  | BirdLife International. 2019. Larus canus (amended version of 2018 assessment). The IUCN Red List of Threatened Species 2019: e.T22694308A155576460. https://dx.doi.org/10.2305/IUCN.UK.2018-2.RLTS.T22694308A155576460.en. Downloaded on 01 September 2021. |
| *Larus marinus* | 690 000 – 940 000 |  | BirdLife International. 2018. Larus marinus. The IUCN Red List of Threatened Species 2018: e.T22694324A132342572. https://dx.doi.org/10.2305/IUCN.UK.2018-2.RLTS.T22694324A132342572.en. Downloaded on 01 September 2021. |
| *Larus ridibundus* | 4 800 000 – 8 900 000 |  | BirdLife International. 2018. Larus ridibundus. The IUCN Red List of Threatened Species 2018: e.T22694420A132548687. https://dx.doi.org/10.2305/IUCN.UK.2018-2.RLTS.T22694420A132548687.en. Downloaded on 01 September 2021. |
| *Melanitta nigra* | 1 600 000 |  | BirdLife International. 2018. Melanitta nigra. The IUCN Red List of Threatened Species 2018: e.T22724879A132257623. https://dx.doi.org/10.2305/IUCN.UK.2018-2.RLTS.T22724879A132257623.en. Downloaded on 01 September 2021. |
| *Mergellus albellus* | 130 000 |  | BirdLife International. 2016. Mergellus albellus. The IUCN Red List of Threatened Species 2016: e.T22680465A85991357. https://dx.doi.org/10.2305/IUCN.UK.2016-3.RLTS.T22680465A85991357.en. Downloaded on 01 September 2021. |
| *Pelecanus crispus* | 1.5 x population of mature individuals (based on expert advice) | 11 400 – 13 400 | BirdLife International. 2018. Pelecanus crispus (amended version of 2017 assessment). The IUCN Red List of Threatened Species 2018: e.T22697599A122838534. https://dx.doi.org/10.2305/IUCN.UK.2017-3.RLTS.T22697599A122838534.en. Downloaded on 01 September 2021. |
| *Phalacrocorax carbo* | 1 400 000 – 2 100 000 |  | BirdLife International. 2019. Phalacrocorax carbo (amended version of 2018 assessment). The IUCN Red List of Threatened Species 2019: e.T22696792A155523636. https://dx.doi.org/10.2305/IUCN.UK.2018-2.RLTS.T22696792A155523636.en. Downloaded on 01 September 2021. |
| *Philomachus pugnax* | 1 594 000 – 9 940 000 |  | BirdLife International. 2016. Calidris pugnax. The IUCN Red List of Threatened Species 2016: e.T22693468A86591264. https://dx.doi.org/10.2305/IUCN.UK.2016-3.RLTS.T22693468A86591264.en. Downloaded on 01 September 2021. |
| *Podiceps cristatus* | 915 000 – 1 400 000 |  | BirdLife International. 2019. Podiceps cristatus. The IUCN Red List of Threatened Species 2019: e.T22696602A154250080. https://dx.doi.org/10.2305/IUCN.UK.2019-3.RLTS.T22696602A154250080.en. Downloaded on 01 September 2021. |
| *Porzana pusilla* | 1.5 x population of mature individuals (based on expert advice) | 500 000 – 999 999 | BirdLife International. 2019. Zapornia pusilla. The IUCN Red List of Threatened Species 2019: e.T22692667A154256374. https://dx.doi.org/10.2305/IUCN.UK.2019-3.RLTS.T22692667A154256374.en. Downloaded on 01 September 2021. |
