# Supplementary material for: Quantifying Time-Dependent Predictors for the International Spatial Spread of Highly Pathogenic Avian Influenza H5NX: Focus on Trade and Surveillance Efforts
Source: Transbound Emerg Dis. 2025 May 8;2025:2020766. doi: 10.1155/tbed/2020766 (PMC12643678; doi:10.1155/tbed/2020766)
Supplement: Supporting Information 5 — Table S3: Results of final GLMMs regressing HPAI first introduction/reintroduction in the exposed country on quantity of poultry commodities traded, incoming migratory birds, characteristics of exposed and source countries and temporality. [file 2020766.f5.docx]

**Table S3.** Results of final GLMMs regressing HPAI first introduction/reintroduction in the exposed country on quantity of poultry commodities traded, incoming migratory birds, characteristics of exposed and source countries and temporality. Significant associations are highlighted in grey. Associations presented in this table are those from the model selected for each clade (preferred simplest model with lowest AIC value). Predictors with no data in the table (empty cells) are those that have been excluded from the preferred models with lowest AIC values. Predictors with data in the table but without grey highlighting are predictors that were not excluded from the preferred models, but for which associations were tested as non-significant.

|  | **Clade** **2.3.2.1c** | | | **Clade 2.3.4.4b** | | |
| --- | --- | --- | --- | --- | --- | --- |
|  | **AIC=378.4** | | | **AIC=588** | | |
|  | **AUC=** **0.72** | | | **AUC=0.81** | | |
| **Effect** | **OR** | **95%CI** | **p-value** | **OR** | **95%CI** | **p-value** |
| Trade (trade from affected countries, sum centered and scaled quantity) |  |  |  |  |  |  |
| Chicken hatching eggs |  |  |  |  |  |  |
| Chicken lighter than 185g | **1.13** | **1.01, 1.28** | **0.05** | **1.20** | **1.03, 1.40** | **0.01** |
| Chicken of 185g or more |  |  |  |  |  |  |
| Hatching eggs of other poultry | **1.18** | **1.01, 1.39** | **0.03** |  |  |  |
| Other poultry lighter than 185g |  |  |  |  |  |  |
| Other poultry of 185g or more |  |  |  |  |  |  |
| Migratory birds (incoming flow from affected countries, sum centered and scaled index for population size) | 1.20 | 0.96, 1.51 | 0.09 | **1.35** | **1.11, 1.64** | **0.001** |
| Proximity with affected countries (sum 1/ centered and scaled [distance]) | **1.54** | **1.14, 2.10** | **0.004** | **2.01** | **1.44, 2.79** | **<0.001** |
| Characteristics exposed countries |  |  |  |  |  |  |
| GDP_per_capita (centered and scaled) |  |  |  |  |  |  |
| Precautions_at_borders | **0.31** | **0.17, 0.58** | **<0.001** | **0.54** | **0.33, 0.88** | **0.01** |
| Characteristics of connected source countries affected by HPAI |  |  |  |  |  |  |
| Passive surveillance in poultry (percentage of countries implementing measure) |  |  |  |  |  |  |
| Active surveillance in poultry (percentage of  countries implementing measure) |  |  |  |  |  |  |
| Surveillance in wild birds (percentage of  countries implementing measure) |  |  |  |  |  |  |
| Preventive vaccination in poultry (percentage of countries implementing measure) |  |  |  |  |  |  |
| GDP_per_capita (average centered and scaled) |  |  |  |  |  |  |
| Time |  |  |  |  |  |  |
| 2009 |  |  |  |  |  |  |
| 2010 (vs. first year of wave) |  |  |  |  |  |  |
| 2011 (vs. first year of wave) |  |  |  |  |  |  |
| 2012 (vs. first year of wave) |  |  |  |  |  |  |
| 2013 (vs. first year of wave) |  |  |  |  |  |  |
| 2014 (vs. first year of wave) |  |  |  |  |  |  |
| 2015 (vs. first year of wave) |  |  |  |  |  |  |
| 2016 (vs. first year of wave) |  |  |  |  |  |  |
| 2017 (vs. first year of wave) |  |  |  |  |  |  |
| 2018 (vs. first year of wave) |  |  |  |  |  |  |
| Quarter2 (vs. quarter 1) | 1.03 | 0.49, 2.17 | 0.91 | 0.60 | 0.30, 1.20 | 0.15 |
| Quarter3 (vs. quarter 1) | **0.21** | **0.06, 0.71** | **0.01** | **0.42** | **0.17, 1.00** | **0.05** |
| Quarter4 (vs. quarter 1) | 1.18 | 0.54, 2.60 | 0.66 | **1.72** | **1.04, 2.85** | **0.03** |
