# Supplementary material for: Quantifying Time-Dependent Predictors for the International Spatial Spread of Highly Pathogenic Avian Influenza H5NX: Focus on Trade and Surveillance Efforts
Source: Transbound Emerg Dis. 2025 May 8;2025:2020766. doi: 10.1155/tbed/2020766 (PMC12643678; doi:10.1155/tbed/2020766)
Supplement: Supporting Information 6 — Table S4: Results of GLM coupled with DTA regressing HPAI spread between countries on quantity of poultry commodities traded, incoming migratory birds and characteristics of exposed and source countries. [file 2020766.f6.docx]

**Table S4**. Results of GLM coupled with DTA regressing HPAI spread between countries on quantity of poultry commodities traded, incoming migratory birds and characteristics of exposed and source countries. Variables with Bayes factor adjusted (BF _adjusted_) greater than 25 are highlighted in grey.

**Clade 2.3.2.1c**

| **Effect** | **Bayes Factor (default)** | **Posterior inclusion probability**  **(default)** | **Bayes Factor (adjusted)** | **Prior inclusion probability**  **(adjusted)** | **Effect Size** | **Effect Size (lower and upper estimates)** |
| --- | --- | --- | --- | --- | --- | --- |
| Trade (centered and scaled sum quantity) |  |  |  |  |  |  |
| Chicken hatching eggs | 0 | 0 | 0 | 0 | 0 | 0, 0 |
| Chicken lighter than 185g | 0.03 | 0 | 0.27 | 0 | -3.45 | 3.45, 3.45 |
| Chicken of 185g or more | 0.03 | 0 | 0.27 | 0 | 0;13 | 0.13, -0.13 |
| Hatching eggs of other poultry | 0.03 | 0 | 0.27 | 0 | -3.13 | -3.13, -3.13 |
| Other poultry lighter than 185g | 0.08 | 0 | 0.81 | 0 | -0.57 | -1.06, -0.04 |
| Other poultry of 185g or more | 0.03 | 0 | 0.09 | 0.01 | -0.01 | -0.01, -0.01 |
| Migratory birds (centered and scaled sum index for population size) | 0.06 | 0 | 0.54 | 0 | 0.06 | 0.01, 0.01 |
| Distance (centered and scaled) | **16143.49** | **1** | **153874** | **0** | **-2.24** | **-3.15, --1.42** |
| Characteristics importing countries |  |  |  |  |  |  |
| GDP_per_capita (centered and scaled) | 0.14 | 0.01 | 1.35 | 0 | 0.22 | 0.14, 0.45 |
| Precautions_at_borders | 0.08 | 0 | 0.81 | 0 | 0.4 | -0.28, -1.49 |
| Characteristics exporting countries |  |  |  |  |  |  |
| Passive surveillance in poultry | 12.31 | 0.37 | 23 | 0.02 | -1.68 | -2.74, --0.54 |
| Active surveillance in poultry | 0.28 | 0.01 | 0.38 | 0.03 | 1.47 | 0.51, 2.98 |
| Surveillance in wild birds | 6.29 | 0.23 | 9.74 | 0.03 | 2.24 | 0.82, 4.26 |
| GDP_per_capita (centered and scaled) | 0.06 | 0 | 0.06 | 0.04 | 0.45 | 0.43, 0.46 |

**Clade 2.3.4.4b**

| **Effect** | **Bayes Factor (default)** | **Posterior inclusion probability**  **(default)** | **Bayes Factor (adjusted)** | **Prior inclusion probability**  **(adjusted)** | **Effect Size** | **Effect Size (lower and upper estimates)** |
| --- | --- | --- | --- | --- | --- | --- |
| Trade (centered and scaled sum quantity) |  |  |  |  |  |  |
| Chicken hatching eggs | **1.13** | **0.04** | **73.51** | **0** | **0.15** | **0.08, 0.27** |
| Chicken lighter than 185g | 1.79 | 0.06 | 23.15 | 0 | 0.11 | 0.04, 0.16 |
| Chicken of 185g or more | 0.09 | 0 | 1.18 | 0 | 0.06 | 0.05, 0.08 |
| Hatching eggs of other poultry | 0.23 | 0.01 | 4.93 | 0 | -0.79 | -1.27, -0.11 |
| Other poultry lighter than 185g | 0.18 | 0.01 | 5.91 | 0 | 0.08 | 0.05, 0.11 |
| Other poultry of 185g or more | 0.89 | 0.03 | 28.82 | 0 | 0.1 | -0.18, 0.19 |
| Migratory birds (centered and scaled sum index for population size) | 0.09 | 0 | 5.89 | 0 | 0.1 | 0.05, 0.15 |
| Distance (centered and scaled) | **15253.52** | **1** | **494450** | **0** | **-1.14** | **-1.6, -0.67** |
| Characteristics importing countries |  |  |  |  |  |  |
| GDP_per_capita (centered and scaled) | **0.74** | **0.03** | **48.31** | **0** | **-0.40** | **-0.69, -0.18** |
| Precautions_at_borders | 0.14 | 0.01 | 4.42 | 0 | -0.26 | -0.82, 0.59 |
| Characteristics exporting countries |  |  |  |  |  |  |
| Passive surveillance in poultry | 7.88 | 0.23 | 17.98 | 0.02 | 1.21 | 0.57, 2.14 |
| Active surveillance in poultry | **20** | **0.43** | **91.98** | **0.01** | **1.63** | **0.47, 2.81** |
| Surveillance in wild birds | 1.04 | 0.04 | 5.56 | 0.01 | 0.58 | -1.42, 2.04 |
| GDP_per_capita (centered and scaled) | **6.85** | **0.21** | **63.22** | **0** | **-0.85** | **-1.43, -0.26** |
