# Supplementary material for: Quantifying Time-Dependent Predictors for the International Spatial Spread of Highly Pathogenic Avian Influenza H5NX: Focus on Trade and Surveillance Efforts
Source: Transbound Emerg Dis. 2025 May 8;2025:2020766. doi: 10.1155/tbed/2020766 (PMC12643678; doi:10.1155/tbed/2020766)
Supplement: Supporting Information 7 — Table S5: Counts of quarters, countries, and sequences for each model and clade. [file 2020766.f7.docx]

**Table S5**. Counts of quarters, countries and viral strains for each model and clade.

| **Clade** | **Model** | **Count of quarters** | **Count of countries** | | | | | **Number of sequences** | | | | |
| --- | --- | --- | --- | --- | --- | --- | --- | --- | --- | --- | --- | --- |
|  |  |  | **All** | **Africa** | **Americas** | **Asia** | **Europe** | **All** | **Africa** | **Americas** | **Asia** | **Europe** |
| 2.3.2.1c | GLMM | 35 | 31 | 7 | 1 | 20 | 3 |  |  |  |  |  |
|  | Phylogeography-informed GLM |  | 23 | 7 | 0 | 13 | 3 | 261 | 35 | 0 | 220 | 6 |
| 2.3.4.4b | GLMM | 21 | 55 | 9 | 0 | 16 | 30 |  |  |  |  |  |
|  | Phylogeography-informed GLM |  | 28 | 7 | 0 | 5 | 16 | 254 | 23 | 0 | 48 | 183 |
