# Supplementary material for: Quantifying Time-Dependent Predictors for the International Spatial Spread of Highly Pathogenic Avian Influenza H5NX: Focus on Trade and Surveillance Efforts
Source: Transbound Emerg Dis. 2025 May 8;2025:2020766. doi: 10.1155/tbed/2020766 (PMC12643678; doi:10.1155/tbed/2020766)
Supplement: Supporting Information 8 — Table S6: Comparative results of a) the GLMM for disease introduction and reintroduction, and b) phylogeography-informed GLM. [file 2020766.f8.docx]

**Table S6**. shows comparative results of a) the GLMM for disease introduction and reintroduction, and b) phylogeography-informed GLM. Non-significant associations were marked as “Not significant”. Association with BF_adjusted_ lower than 25 were marked as “Not found”. Other associations are highlighted as “positive” or “negative”.

|  | **Clade 2.3.2.1c** | | **Clade 2.3.4.4b** | |
| --- | --- | --- | --- | --- |
| **Effect** | **GLMM** | **Phylogeography-informed GLM** | **GLMM** | **Phylogeography-informed GLM** |
| Trade |  |  |  |  |
| Chicken hatching eggs | Not significant | Not significant | Not found | **Positive** |
| Chicken lighter than 185g | **Positive** | Not significant | **Positive** | Not found |
| Chicken of 185g or more | Not significant | Not significant | Not found | Not found |
| Hatching eggs of other poultry | **Positive** | Not significant | Not found | Not found |
| Other poultry lighter than 185g | Not significant | Not significant | Not found | Not found |
| Other poultry of 185g or more | Not significant | Not significant | Not found | Not found |
| Migratory birds | Not significant | Not significant | **Positive** | Not found |
| Proximity | **Positive** | *Not tested* | **Positive** | *Not tested* |
| Distance | *Not tested* | **Negative** | *Not tested* | **Negative** |
| Characteristics exposed countries |  |  |  |  |
| GDP_per_capita | Not significant | Not significant | Not found | **Negative** |
| Precautions_at_borders | **Negative** | Not significant | **Negative** | Not found |
| Characteristics exporting countries |  |  |  |  |
| Passive surveillance in poultry | Not significant | Not significant | Not found | Not found |
| Active surveillance in poultry | Not significant | Not significant | Not found | **Positive** |
| Surveillance in wild birds | Not significant | Not significant | Not found | Not found |
| Preventive vaccination in poultry | Not significant | *Not tested* | Not found | *Not tested* |
| GDP_per_capita | Not significant | Not significant | Not found | **Negative** |
| Time |  |  |  |  |
| Year | Not significant | *Not tested* | Not found | *Not tested* |
| Quarter 2 (vs. quarter 1) | Not significant | *Not tested* | Not found | *Not tested* |
| Quarter 3 (vs. quarter 1) | **Negative** | *Not tested* | **Negative** | *Not tested* |
| Quarter 4 (vs. quarter 1) | Not significant | *Not tested* | **Positive** | *Not tested* |
