# Supplementary material for: Quantifying Time-Dependent Predictors for the International Spatial Spread of Highly Pathogenic Avian Influenza H5NX: Focus on Trade and Surveillance Efforts
Source: Transbound Emerg Dis. 2025 May 8;2025:2020766. doi: 10.1155/tbed/2020766 (PMC12643678; doi:10.1155/tbed/2020766)
Supplement: Supporting Information 9 — Section S1: Detailed description of the GLMM for disease introduction and reintroduction. [file 2020766.f9.docx]

**Section S1.** Detailed description of the generalized Linear Mixed Model (GLMM) for disease introduction and reintroduction

For each clade, the following GLMM was used to predict HPAI introduction in a country during a quarter:

$\boldsymbol{logit}\left( \boldsymbol{P}_{\boldsymbol{i,t}} \right)\boldsymbol{=}\boldsymbol{\beta}_{\boldsymbol{0}}\boldsymbol{+}\boldsymbol{\beta}_{\boldsymbol{1}}\boldsymbol{Y}_{\boldsymbol{t}}\boldsymbol{+}\boldsymbol{\beta}_{\boldsymbol{2}}\boldsymbol{Trim}_{\boldsymbol{t}}\boldsymbol{+}{\boldsymbol{\beta}_{\boldsymbol{3}}\boldsymbol{HEC}}_{\boldsymbol{i,t}}\boldsymbol{+}{\boldsymbol{\beta}_{\boldsymbol{4}}\boldsymbol{HEOP}}_{\boldsymbol{i,t}}\boldsymbol{+}{\boldsymbol{\beta}_{\boldsymbol{5}}\boldsymbol{LC}}_{\boldsymbol{i,t}}\boldsymbol{+}{\boldsymbol{\beta}_{\boldsymbol{6}}\boldsymbol{LOP}}_{\boldsymbol{i,t}}\boldsymbol{+}{\boldsymbol{\beta}_{\boldsymbol{7}}\boldsymbol{HC}}_{\boldsymbol{i,t}}\boldsymbol{+}{\boldsymbol{\beta}_{\boldsymbol{8}}\boldsymbol{HOP}}_{\boldsymbol{i,t}}\boldsymbol{+}{\boldsymbol{\beta}_{\boldsymbol{9}}\boldsymbol{MB}}_{\boldsymbol{i,t}}\boldsymbol{+}\boldsymbol{\beta}_{\boldsymbol{10}}\boldsymbol{GDP}_{\boldsymbol{i,t}}\boldsymbol{+}\boldsymbol{\beta}_{\boldsymbol{11}}\boldsymbol{PB}_{\boldsymbol{i,t}}\boldsymbol{+}\boldsymbol{\beta}_{\boldsymbol{12}}\boldsymbol{mGD}\boldsymbol{P}_{\boldsymbol{i,t}}\boldsymbol{+}\boldsymbol{\beta}_{\boldsymbol{13}}{\boldsymbol{Prox}_{\boldsymbol{i,t}}\boldsymbol{+}\boldsymbol{\beta}_{\boldsymbol{14}}\boldsymbol{P}}_{\left( \boldsymbol{ACT} \right)\boldsymbol{j,t}}\boldsymbol{+}\boldsymbol{\beta}_{\boldsymbol{15}}\boldsymbol{P}_{\left( \boldsymbol{PAS} \right)\boldsymbol{j,t}}\boldsymbol{+}{\boldsymbol{\beta}_{\boldsymbol{16}}\boldsymbol{P}}_{\left( \boldsymbol{WILD} \right)\boldsymbol{j,t}}\boldsymbol{+}{\boldsymbol{\beta}_{\boldsymbol{17}}\boldsymbol{P}}_{\left( \boldsymbol{VAC} \right)\boldsymbol{j,t}}\boldsymbol{+}\boldsymbol{B}_{\boldsymbol{i}}\boldsymbol{+}\boldsymbol{\epsilon}_{\boldsymbol{i,t}}$ (1)

With

- $logit\left( P_{i,t} \right)=log\left( \frac{P_{i,t}}{1-P_{i,t}} \right)$
- $P_{i,t}$ being the probability of HPAI introduction for the country $i$ at the time $t$.
- With $\beta_{x}$ the parameters of the model. Except for $\beta_{0}$, exp($\beta_{x})$ is the OR adjusted of the corresponding factor
- $Y_{t}$being the year of the HPAI introduction
- ${Trim}_{t}$ being the quarter of the HPAI introduction
- ${HEC}_{i,t}$ being the quantity of chicken hatching eggs imported by $i$ from all affected countries, at the time $t$
- ${HEOP}_{i,t}$ being the quantity of hatching eggs of other poultry imported by $i$ from all affected countries at the time $t$
- ${LC}_{i,t}$ being the quantity of chicken lighter than 185g imported by $i$ from all affected countries at the time $t$
- ${LOP}_{i,t}$ being the quantity of other poultry lighter than 185g imported by $i$ from all affected countries at the time $t$
- ${HC}_{i,t}$ being the quantity of chicken of 185g or more imported by $i$ from all affected countries at the time $t$
- ${HOP}_{i,t}$ being the quantity of other poultry of 185g or more imported by $i$ from all affected countries at the time $t$
- ${MB}_{i,t}$ being the proxy for total migratory bird population introduced in $i$ from all affected countries at the time $t$
- ${GDP}_{i,t}$ being the GDP per capita of the country $i$ at the time $t$.
- ${PB}_{i,t}$ being the implementation of precautions at borders in the country $i$ at the time $t$
- $mGDP_{i,t}=\frac{1}{n}\sum_{j=1}^{n} {GDP}_{j,t}$ being the average GDP per capita of all other affected countries $j$ at the time $t$. $n$ being the total number of all other affected countries $j$ at the time $t$.
- ${Prox}_{i,t}=\sum_{j=1}^{n} \frac{1}{{DIS}_{i,j}}$ being the proximity of country $i$ with all affected countries at the time $t$. It is calculated as the sum of inverses of the centroid-centroid distance between $i$ and each affected country $j$; proximity between countries captures informal trade, wild birds’ movements across short distances and human movements.
- $P_{\left( ACT \right)j,t}$ being the percentage of countries among all other affected countries at the time $t$ reporting active surveillance in poultry
- $P_{\left( PAS \right)j,t}$ being the percentage of countries among all other affected countries at the time $t$ reporting passive surveillance in poultry
- $P_{\left( WILD \right)j,t}$ being the percentage of countries among all other affected countries at the time $t$ reporting surveillance in wild birds; and
- $P_{\left( VAC \right)j,t}$ being the percentage of countries among all other affected countries at the time $t$ reporting preventive vaccination in poultry during t
- $B_{i}$ the random effect of the country $i$ on $\beta_{0}$
- $\epsilon_{i,t}$ the residual error

And year “$Y$”, quarter “$Trim$” and importing country “$i$” being treated as qualitative variables, precautions at borders “$PB$ ” as binomial variable and all explanatory variables as quantitative variables.
