# Supplementary material for: Quantifying Time-Dependent Predictors for the International Spatial Spread of Highly Pathogenic Avian Influenza H5NX: Focus on Trade and Surveillance Efforts
Source: Transbound Emerg Dis. 2025 May 8;2025:2020766. doi: 10.1155/tbed/2020766 (PMC12643678; doi:10.1155/tbed/2020766)
Supplement: Supporting Information 10 — Section S2: Detailed description of the GLM coupled with phylogeographic inference with time-dependent coefficients for viral spread. [file 2020766.f10.docx]

**Section S2.** Detailed description of the Generalized Linear Model (GLM) coupled with phylogeographic inference with time dependent coefficients for viral spread

Bayesian phylogeographic inference models discrete diffusion as a continuous-time Markov chain process parameterized in terms of a $\boldsymbol{KxK}$ infinitesimal rate matrix $\boldsymbol{\Lambda}$ discrete location change with $\boldsymbol{K}$ representing the number of location states. The GLM diffusion model extends this by adopting a GLM approach that takes an arbitrary number $\boldsymbol{P}$ of predictors $\boldsymbol{X=(}\boldsymbol{x}_{\boldsymbol{1}}\boldsymbol{,\ldots}\boldsymbol{x}_{\boldsymbol{p}}\boldsymbol{)}$**,** where a single predictor $\boldsymbol{x}_{\boldsymbol{p}}$ is a flattened vector of quantities corresponding to entries in the $\boldsymbol{i}$ to $\boldsymbol{j}$ rate matrix $\boldsymbol{x}_{\boldsymbol{p}} \boldsymbol{=(}\boldsymbol{x}_{\boldsymbol{1,2,p}}\boldsymbol{\ldots}\boldsymbol{x}_{\boldsymbol{K-1,K,p}}\boldsymbol{)}$ . The GLM considers every instantaneous movement rate $\boldsymbol{\Lambda}_{\boldsymbol{ij}}$ for $\boldsymbol{i\neq}$ $\boldsymbol{j}$ in $\boldsymbol{\Lambda}$ as a log linear function of the set of predictors $\boldsymbol{X}$.The following variables were included as potential time-variable predictors, with the first two predictors included for testing the impact of sampling effects (Lemey et al., 2014):

$\boldsymbol{log}\left( \boldsymbol{\Lambda}_{\boldsymbol{i,j}} \right)\boldsymbol{=}{\boldsymbol{\beta}_{\boldsymbol{1}}\boldsymbol{\delta}_{\boldsymbol{1}}\boldsymbol{nj} \mathbf{+}{\boldsymbol{\beta}_{\boldsymbol{2}}\boldsymbol{\delta}_{\boldsymbol{2}}\boldsymbol{ni} \mathbf{+}\boldsymbol{\beta}}_{\boldsymbol{3}}\boldsymbol{\delta}_{\boldsymbol{3}}\boldsymbol{HEC}}_{\boldsymbol{ji,t}}\boldsymbol{+}{\boldsymbol{\beta}_{\boldsymbol{4}}\boldsymbol{\delta}_{\boldsymbol{4}}\boldsymbol{HEOP}}_{\boldsymbol{ji,t}}\boldsymbol{+}{\boldsymbol{\beta}_{\boldsymbol{5}}\boldsymbol{\delta}_{\boldsymbol{5}}\boldsymbol{LC}}_{\boldsymbol{ji,t}}\boldsymbol{+}{\boldsymbol{\beta}_{\boldsymbol{6}}\boldsymbol{\delta}_{\boldsymbol{6}}\boldsymbol{LOP}}_{\boldsymbol{ji,t}}\boldsymbol{+}{\boldsymbol{\beta}_{\boldsymbol{7}}\boldsymbol{\delta}_{\boldsymbol{7}}\boldsymbol{HC}}_{\boldsymbol{ji,t}}\boldsymbol{+}{\boldsymbol{\beta}_{\boldsymbol{8}}\boldsymbol{\delta}_{\boldsymbol{8}}\boldsymbol{HOP}}_{\boldsymbol{ji,t}}\boldsymbol{+}{\boldsymbol{\beta}_{\boldsymbol{9}}\boldsymbol{\delta}_{\boldsymbol{9}}\boldsymbol{MB}}_{\boldsymbol{ji,t}}\boldsymbol{+}\boldsymbol{\beta}_{\boldsymbol{10}}{\boldsymbol{\delta}_{\boldsymbol{10}}\boldsymbol{GDP}}_{\boldsymbol{i,t}}\boldsymbol{+}\boldsymbol{\beta}_{\boldsymbol{11}}\boldsymbol{\delta}_{\boldsymbol{11}}\boldsymbol{PB}_{\boldsymbol{i,t}}\boldsymbol{+}\boldsymbol{\beta}_{\boldsymbol{12}}\boldsymbol{\delta}_{\boldsymbol{12}}\boldsymbol{GD}\boldsymbol{P}_{\boldsymbol{j,t}}\boldsymbol{+}\boldsymbol{\beta}_{\boldsymbol{13}}\boldsymbol{\delta}_{\boldsymbol{13}}\boldsymbol{D}{\boldsymbol{i}\boldsymbol{s}_{\boldsymbol{i,j}}\boldsymbol{+}\boldsymbol{\beta}_{\boldsymbol{14}}\boldsymbol{\delta}_{\boldsymbol{14}}\boldsymbol{ACT}}_{\boldsymbol{j,t}}\boldsymbol{+}\boldsymbol{\beta}_{\boldsymbol{15}}\boldsymbol{\delta}_{\boldsymbol{15}}\boldsymbol{PAS}_{\boldsymbol{j,t}}\boldsymbol{+}{\boldsymbol{\beta}_{\boldsymbol{16}}\boldsymbol{\delta}_{\boldsymbol{16}}\boldsymbol{WILD}}_{\boldsymbol{j,t}}\boldsymbol{+}\boldsymbol{\epsilon}_{\boldsymbol{i,t}}$ (2)

Where $\boldsymbol{\beta=(}\boldsymbol{\beta}_{\boldsymbol{1}}\boldsymbol{,\ldots}\boldsymbol{\beta}_{\boldsymbol{p}}\boldsymbol{)}$**,** represent the effective sizes for the predictors, quantifying their contribution to $\boldsymbol{\Lambda}$, and $\boldsymbol{(}\boldsymbol{\delta}_{\boldsymbol{1}}\boldsymbol{,\ldots}\boldsymbol{,\delta}_{\boldsymbol{p}}\boldsymbol{)}$ are (0,1)-indicator variables that govern the inclusion or exclusion of the predictors in the model.

And

- $nj$ being the number of samples from the origin location *j*
- $ni$ being the number of samples from destination location *i*
- ${HEC}_{ji,t}$ being the quantity of chicken hatching eggs imported by $i$ from *j*, at the time $t$
- ${HEOP}_{ji,t}$ being the quantity of hatching eggs of other poultry imported by $i$ from *j* at the time $t$
- ${LC}_{ji,t}$ being the quantity of chicken lighter than 185g imported by $i$ from *j* at the time $t$
- ${LOP}_{ji,t}$ being the quantity of other poultry lighter than 185g imported by $i$ from *j* at the time $t$
- ${HC}_{ji,t}$ being the quantity of chicken of 185g or more imported by $i$ from *j* at the time $t$
- ${HOP}_{ji,t}$ being the quantity of other poultry of 185g or more imported by $i$ from *j* at the time $t$
- ${MB}_{ji,t}$ being the proxy for total migratory bird population introduced in $i$ from *j* at the time $t$
- ${GDP}_{i,t}$ being the GDP per capita of the country $i$ at the time $t$.
- ${PB}_{i,t}$ being the implementation of precautions at borders in the country $i$ at the time $t$
- ${GDP}_{j,t}$ being the GDP per capita of the country $j$ at the time $t$.
- ${Dis}_{i,j}$ being the distance of country $i$ with country *j*. It is calculated as the centroid-centroid distance between $i$ and $j$; proximity between countries captures informal trade, wild birds’ movements across short distances and human movements.
- ${ACT}_{j,t}$ being the reporting of active surveillance in poultry of the country $j$ at the time $t$.
- ${PAS}_{j,t}$being the reporting of passive surveillance in poultry of the country $j$ at the time $t.$
- ${WILD}_{j,t}$being the reporting surveillance in wild birds of the country $j$ at the time $t.$
- $\epsilon_{i,t}$ being the residual error
